# Immune Modulation by Personalized vs Standard Prehabilitation Before Major Surgery: A Randomized Clinical Trial

**DOI:** 10.1001/jamasurg.2025.4917

**Published:** 2025-11-12

**Authors:** Amélie Cambriel, Amy Tsai, Benjamin Choisy, Maximilian Sabayev, Julien Hedou, Elizabeth Shelton, Kreeti Singh, Jonas Amar, Valentin Badea, Serena Bruckman, Ed Ganio, Jakob Einhaus, Dorien Feyaerts, Ina Stelzer, Masaki Sato, Olivier Langeron, T. Adam Bonham, Dyani Gaudillière, Andrew Shelton, Cindy Kin, Brice Gaudillière, Franck Verdonk

**Affiliations:** 1 Department of Anesthesiology, Perioperative and Pain Medicine, Stanford University School of Medicine, Stanford, California; 2 Department of Anesthesiology and Critical Care, APHP, Hopital Saint Antoine, DMU DREAM, AP-HP, Paris, France; 3 UMRS_938, Centre de Recherche Saint-Antoine (CRSA), Sorbonne Université-Inserm, Paris, France; 4 Department of Surgery, Stanford University School of Medicine, Stanford, California; 5 SurgeCare, SAS, Paris, France; 6 Institute of Computational and Mathematical Engineering, Stanford University, Stanford, California; 7 Department of Pathology and Neuropathology, University Hospital and Comprehensive Cancer Center Tübingen, Tübingen, Germany; 8 Department of Pathology, University of California, San Diego, La Jolla; 9 Department of Anesthesiology and Critical Care, CHU Brest, Brest, France

## Abstract

**Importance:**

Prehabilitation programs are increasingly recognized for their potential to improve surgical outcomes. However, their efficacy remains debated, largely due to a lack of pathophysiologically driven implementation and limited personalization.

**Objective:**

To determine the impact of personalized vs standard prehabilitation on preoperative physical, cognitive, and immune function and postoperative outcomes.

**Design, Setting, and Participants:**

In this prospective, single-blinded, randomized interventional trial conducted from June 2020 to September 2022 in a single academic medical center, 58 patients undergoing major elective surgery were randomized to standard (n = 30) or personalized prehabilitation (n = 28) using block randomization. Those with contraindication to exercise, an American Society of Anesthesiologists score 4 or higher, in palliative care, less than 14 days between screening and surgery were excluded. Data were analyzed from April 2023 to May 2025.

**Intervention:**

The personalized group received 2 weekly one-on-one remote coaching sessions tailored to individual progress in 4 domains (physical activity, nutrition, cognitive training, and mindfulness), whereas the standard group followed a paper-based program, including the same domains, without individualized support.

**Main Outcomes and Measures:**

Primary clinical outcomes included cognitive assessments and physical performance measures, including the wall squat test, timed-up-and-go test, and 6-minute walk test (6MWT). The primary immunological outcomes included major innate and adaptive immune cell frequencies and intracellular signaling responses measured using a 47-plex mass cytometry immunoassay.

**Results:**

Of 58 patients (median [IQR] age, 57 [45-67] years; 31 [57%] female) enrolled, 54 completed the study (n = 27 per group). The personalized group exhibited significant improvements in physical measures (eg, median [IQR] 6MWT: 496 [340-619] minutes before prehab versus 546 [350-728] minutes after; *P* = .03) and fewer moderate-to-severe postoperative complications (4 vs 11 Clavien-Dindo grade >1; *P* = .04). Multivariable modeling identified profound and cell-type specific immune alterations postprehabilitation compared to baseline (area under the receiver operating characteristic curve [AUROC], 0.88; 0.79-0.97; *P* < .001; leave-one-out cross-validation), including dampened phosphorylated protein kinase R-like endoplasmic reticulum kinase 1/2 signaling in classical monocytes and myeloid-derived suppressor cells after interleukin 2, 4, and 6 stimulation, and reduced phosphorylated cyclic adenosine monophosphate response–element binding protein signaling in Th1 cells. In contrast, the standard group showed only moderate clinical improvements and no immune changes (AUROC = 0.63; 95% CI, 0.48-0.78; *P* = .12).

**Conclusions and Relevance:**

In this study, personalized prehabilitation significantly altered the immunome before surgery, dampening inflammatory signaling responses previously implicated in the pathophysiology of key surgical outcomes, including surgical site infections and postoperative neurocognitive decline. These changes were accompanied by improved physical and cognitive function before surgery and decreased postoperative complications. These findings support the use of personalized prehabilitation and provide an avenue for biologically driven monitoring of prehabilitation efficacy, and individual tailoring of programs to optimize surgical readiness and recovery.

**Trial Registration:**

ClinicalTrials.gov Identifier: NCT04498208

## Key Points

**Question:**

How do personalized prehabilitation programs modulate the peripheral immune system in patients undergoing major elective surgery?

**Findings:**

In this randomized clinical trial, 58 patients scheduled for major elective surgery received either personalized or standard prehabilitation. High-dimensional immune profiling with mass cytometry revealed profound and cell type-specific dampening of proinflammatory signaling responses in the personalized prehabilitation group but not in the standard group; patients in the personalized prehabilitation group also showed significant improvements in both physical and cognitive function, with significantly fewer severe postoperative complications.

**Meaning:**

The findings in this study suggest that tailored interventions may optimize surgical preparedness and reduce complications.

## Teaser

This randomized clinical trial evaluates preoperative physical, cognitive, and immune function and postoperative outcomes following personalized vs standard prehabilitation programs implemented before major surgery.

## Introduction

Up to 30% of patients undergoing major elective surgery experience postoperative complications,^1,2^ leading to delayed functional recovery, prolonged hospital stays,^3,4^ and considerable health care costs,^4^ exceeding $145 billion annually in the US.^5^

In recent decades, the adoption of enhanced recovery after surgery (ERAS) protocols has significantly improved surgical outcomes via interventions from the day before surgery to hospital discharge.^6^ By contrast, prehabilitation focuses on enhancing patients’ physiological reserve weeks to months before surgery to improve their resilience to surgical injury.^7^ Early prehabilitation programs focused on physical exercise^8^ whereas more recent multimodal approaches, including some combination of exercise, nutrition, cognitive training, and stress reduction, have shown more promise in improving surgical recovery.^7,9,10,11^ Amidst the growing body of literature on prehabilitation, the true impact on postoperative outcomes remains debated. Meta-analyses of studies conducted between 2000 and 2018 report conflicting results, particularly regarding postoperative complications and length of stay.^10,12,13,14,15,16,17^ One key reason for this variability is the heterogeneity of prehabilitation programs, the degree of personalization and coaching, and patient selection criteria.^10,12^ In a health care system bound by increasing resource constraints, there is a crucial need to identify patients most likely to benefit from prehabilitation and to adapt programs to their needs.

Current tools for risk stratification, such as the Risk Analysis Index–Administrative, the American College of Surgeons National Surgical Quality Improvement Program surgical risk score, and the Fried frailty score, rely exclusively on clinical data,^18,19,20,21^ and have limited predictive performance. A better understanding of the pathophysiological effect of prehabilitation programs on mechanisms driving surgical recovery is a crucial first step to design effective prehabilitation programs tailored to each patient’s biology.

Surgical tissue injury triggers profound local and peripheral immune responses engaging a network of innate and adaptive immune cells.^22,23,24,25,26,27^ Successful surgical recovery requires a functional balance between proinflammatory and immunosuppressive responses essential for pathogen defense, wound healing, and pain resolution.^28,29,30^ As such, in-depth immune profiling before and after prehabilitation holds promise for identifying modifiable immunologic parameters to inform the design of personalized prehabilitation interventions.

We hypothesized that personalized prehabilitation would have a greater immune modulatory effect than standard prehabilitation, resulting in better postoperative outcomes compared to standard prehabilitation. The primary goal of this clinical trial was to determine the effect of a personalized prehabilitation program on patients’ immunological status before surgery. Secondary objectives included physical and cognitive improvements with prehabilitation and impact on postoperative complications and hospital length of stay.

## Methods

### Trial Design and Ethics Statements

This prospective, single-center, single-blinded, randomized clinical trial compared standard vs personalized prehabilitation in patients undergoing major elective surgery. It was conducted in accordance with the Declaration of Helsinki, approved by the institutional review board of Stanford Health Care, and registered in 2020 on ClinicalTrials.gov (NCT04498208). The study followed the Consolidated Standards of Reporting Trials (CONSORT) reporting guideline. The trial protocol is in Supplement 1. After being informed by their surgeons and the research team, participants provided written consent to the study.

### Participants

Eligible participants were 18 years and older, scheduled for major elective surgery (abdominal, thoracic, plastic and reconstructive, and neurosurgical procedures) under general anesthesia at Stanford Health Care, with surgery planned 14 days or later after enrollment. Major surgery was defined according to the Delphi European Surgical Association consensus as any surgery meeting 1 or more of these criteria: serious patient comorbidity, predicted 30-day morbidity greater than 30%, predicted mortality greater than 2%, and the need for intermediate or intensive care.^31^ Inclusion required fluency in English and ability to provide written informed consent. Exclusion criteria included premorbid conditions or orthopedic impairments contraindicating exercise, cognitive disabilities defined as evolutive neurological or neurodegenerative disease, expected length of stay in the hospital less than 48 hours, American Society of Anesthesiologists (ASA) class 4 or higher, terminal illness under palliative care, illiteracy, legal conservatorship, pregnancy or breastfeeding, and missing blood samples from before or after prehabilitation. The study design is represented in eFigure 2 in Supplement 1.

### Randomization and Blinding

Patients were randomized in blocks of 10, stratified by age, to receive standard or personalized prehabilitation 2 to 6 weeks before surgery. Outcomes assessors, surgeons, anesthesiologists, and statisticians were blinded to group allocation. Patients were not blinded to prehabilitation assignment due to the nature of the intervention.

### Prehabilitation Programs

In the standard prehabilitation group, patients received a printed booklet with guidance on physical exercise, nutrition, stress reduction, and cognitive training. Physical exercise recommendations included 4 difficulty levels of strength and cardio exercises and 7 stretching exercises. Strength exercises included upper body, lower body, and core. Cardio exercise regimens included low-intensity and high-intensity options. The nutritional recommendations followed a Mediterranean-style diet; stress and pain management included the breathe-scan-visualize check-in technique, and cognitive training recommendations included daily use of the Lumosity app (Lumos Labs). After 1 orientation session with a physician member of the research team reviewing the booklet, patients were instructed to select their highest exercise level based on their comfort and received no further coaching.

Patients in the personalized prehabilitation group attended twice-weekly one-on-one remote coaching sessions tailored to their individual abilities and progress, including the same 4 domains as the standard prehabilitation. Patients had access to an online platform (Notion [Notion Labs]) that offered daily meditation recordings, explanations for the exercise program of the week, daily recommendations for healthy meals and Mediterranean-diet recipes, a reminder to practice cognitive training on the Lumosity app, as well as a feedback section where patients could record their challenges to discuss during coaching sessions (eMethods in Supplement 1). Sessions were provided weekly by both a physician (B.C.), who focused on nutrition, cognition, and behavioral strategies, and a physical therapist (S.B.), who specialized in exercise. Initial sessions were dedicated to education and creation of a tailored prehabilitation program. Subsequent sessions were focused on patients’ achievements and resultant adaptations of the prehabilitation plan. To ensure coherence and individualized adaptation, weekly feedback sessions were held with the 2 mentors (B.C. and S.B.) together with clinical trial team members (F.V., C.K., B.G., and D.G.), during which patient adherence, engagement, and progression were discussed to maximize consistency while ensuring tailored support. If surgery was scheduled more than 6 weeks after randomization, patients were asked to continue the program, but no remote coaching sessions were scheduled after 6 weeks.

### Data Collection

Whole-blood collection, for immune single-cell analysis, and physical and cognitive ability assessments occurred at 2 points: baseline (before prehabilitation) and preoperatively (after prehabilitation). A trained member of the research team (E.S.), blinded to group assignment, administered the 6-minute walk test (6MWT), the timed up-and-go test, wall squat test, and Quick Mild Cognitive Impairment (qMCI) test. Adherence was monitored weekly by a blinded evaluator using a 4-point Likert scale (1 = no prehabilitation engagement, 4 = daily adherence; see the eMethods in Supplement 1). Study data were collected and managed using REDCap electronic data capture tools hosted at Stanford University.^32^ The Charlson Comorbidity Index^33^ was automatically computed from electronic health records using REDCap. Collection, processing, and analysis of blood samples by mass cytometry followed previously described protocols^27,35,36^ (eMethods in Supplement 1).

### Study Outcomes

Primary outcomes included the effect of standard vs personalized prehabilitation on improvement in physical and cognitive performance before surgery, 30-day postoperative complications (using the Clavien-Dindo classification^34^) and hospital length of stay, and immunological measures.

### Statistical Analysis

Statistical analyses were performed using Python3 with the Visual Studio Code Interface (Microsoft), and the following libraries: pandas version 2.1.4 (data manipulation),^37^ SciPy version 1.11.4,^38^ NumPy version 1.26.2 (scientific computation),^39^ matplotlib version 3.8.2,^40^ seaborn version 0.13.0 (data visualization),^41^ and scikit-learn version 1.3.2 (machine learning).^42^ Analysis code and datasets are available on request.

#### Power Analysis

Power calculations to distinguish between preintervention and postintervention immune marker profiles within each treatment group indicated a sample size of 29 patients per group was required, based on a balanced binary classification task using an area under the receiver operating characteristic curve (AUROC) estimation of 0.85, a confidence interval width of 0.25, a 90% confidence level, and an expected 5% loss to follow-up.

#### Analysis of Clinical Outcomes

Patients’ clinical and demographic characteristics were reported as medians with IQRs for continuous variables and as counts with percentages for categorical variables. Variables were compared using a Mann-Whitney *U* test. If there were more than 2 groups, the Kruskal-Wallis test was used. We assessed within-group changes in physical, cognitive, and immune function following prehabilitation. Comparisons of before vs after prehabilitation scores in each group were performed using a Wilcoxon signed-rank test for the qMCI, 6MWT, timed-up-and-go test, and wall squat test. Clinical data were treated as complete case analysis.

Weekly adherence scores were averaged over the prehabilitation period and compared between groups using the Mann-Whitney *U* test. Postoperative complications within 30 days (Clavien-Dindo classification) and hospital length of stay between groups were also compared with a Mann-Whitney *U* test. If patients presented with more than 1 complication, the most severe complication grade was used to compare grade severity between groups.

#### Immune Profile Classification

Following preprocessing and visualization of the single-cell proteomic dataset (eMethods in Supplement 1), we applied a multivariable modeling approach to identify differences in patients’ immune profiles encompassing endogenous intracellular signaling activities, signaling responses to extracellular stimulations, and immune cell frequency before and after the prehabilitation regimen for each group. To identify a set of distinguishing immune features between time points (before and after prehabilitation), the Stabl algorithm (eMethods in Supplement 1)^43^ was used to fit a classification model. To evaluate model performance on unseen patients, we used a leave-one-patient-out cross-validation procedure: at each step, all biological variables from one patient were completely excluded during training. The model was trained on the remaining patients and evaluated on the held-out individual. This strategy was specifically tailored for multiomic data, where each patient contributes multiple molecular layers. Within each training fold, we performed an inner cross-validation loop to optimize the integration of multiomic signals. This framework allowed to estimate patient-level performance metrics, including AUROC (eMethods in Supplement 1). Data were analyzed from April 2023 to May 2025. Statistical significance was calculated via Mann-Whitney *U* test, with a *P* value less than .05 considered statistically significant.

## Results

### Study Population

**Figure 1.**
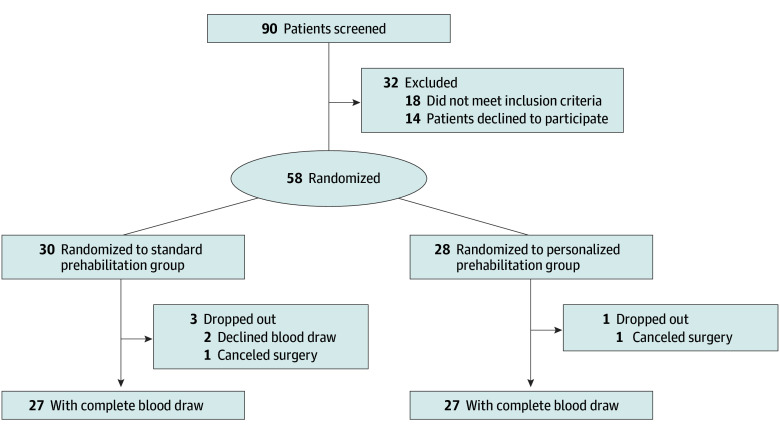
CONSORT Flow Diagram

Fifty-eight patients (median [IQR] age, 57 [45-67] years; 31 [57%] female and 23 [43%] male) undergoing major elective surgery were randomized to receive either standard (n = 30) or personalized (n = 28) prehabilitation 2 to 6 weeks before surgery, between June 2020 and September 2022 (Figure 1; study design in eFigure 2 in Supplement 1). Three patients in the standard group and 1 in the personalized group withdrew, resulting in 27 patients per group with complete paired blood samples for analysis (Figure 1). Baseline characteristics (Table) were comparable between groups in terms of age, sex, and ASA score. Most patients (n = 43 [79.6%]) underwent abdominal surgery (19 in the standard group and 24 in the personalized group; *P* = .05), and 27 (50.0%) had cancer in both groups.

**Table.** Demographic Characteristics of the Study Population

Characteristic	All (N = 54)	Prehabilitation	
		Standard (n = 27)	Personalized (n = 27)
Age, median (IQR) y	57.0 (45.0-66.8)	52.0 (45.0-69.0)	60.0 (45.0-66.0)
Sex, No. (%)			
Female	31 (57.0)	15 (55.6)	16 (59.3)
Male	23 (43.0)	12 (44.4)	11 (40.7)
BMI, median (IQR)	25.1 (22.3-30.4)	25.1 (22.3-30.1)	24.5 (22.6-30.8)
ASA class, No. (%)			
I	2 (3.7)	1 (3.7)	1 (3.7)
II	23 (43.0)	8 (29.6)	15 (55.6)
III	28 (51.9)	17 (63.0)	11 (40.7)
IV	0	0	0
Surgery type			
Abdominal surgery, No. (%)	43 (79.6)	19 (70.4)	24 (88.9)
Obesity surgery (sleeve or bypass), No.	8	4	4
Colectomy, No.	15	4	11
Abdominal debulking, No.	13	8	5
Pancreatic surgery, No.	2	1	1
Esophagectomy, No.	2	1	1
Ileostomy take down/appendectomy, No.	4	2	2
Urologic procedure, No. (%)	3 (5.6)	3 (11.1)	0
Prostatectomy, No.	1	1	0
Cystectomy, No.	1	1	0
Kidney transplant, No.	1	1	0
Thoracic (lobectomy), No. (%)	2 (3.7)	2 (7.4)	0
Major maxillofacial surgery, No. (%)	6 (11.1)	3 (11.1)	3 (11.1)
Charlson Comorbidity Index, median (IQR)	4 (2-5)	4 (2-5)	2 (2-5)
Comorbidity			
Myocardial infarction or ischemic arteriopathy, No. (%)	10 (18.5)	6 (22.2)	4 (14.8)
Congestive heart failure, No. (%)	1 (1.9)	0	1 (3.7)
Chronic pulmonary disease, No. (%)	9 (16.7)	7 (25.9)	2 (7.4)
Diabetes, No. (%)	7 (13.0)	2 (7.4)	5 (18.5)
Kidney disease, No. (%)^a^	5 (9.3)	2 (7.4)	3 (11.1)
eGFR, median (IQR), mL/min/1.73 m^2^	105 (88-116)	94 (74-115)	110 (97-118)
Cancer, localized, No. (%)	27 (50.0)	16 (59.3)	11 (40.7)
Cancer, metastatic, No. (%)	2 (3.7)	0	2 (7.4)
Prehabilitation duration, median (IQR), d	32.5 (23.5-42.0)	35.0 (22.0-42.0)	31.0 (28.0-42.0)

Abbreviations: ASA, American Society of Anesthesiologists; BMI, body mass index (calculated as weight in kilograms divided by height in meters squared); eGFR, estimated glomerular filtration rate.

^a^ Kidney disease in the Charlson Comorbidity Index is defined as creatinine >3 mg/dL, dialysis, or history of kidney transplant.

The median (IQR) prehabilitation duration was 35 (22-42) days in the standard group and 31 (28-42) days in the personalized group (*P* = .89). Overall adherence on a 4-point Likert scale was higher in the personalized group (mean [SD], 3.3 [0.4]) compared to the standard group (mean [SD], 3.0 [0.5]) but not clinically significant. Adherence for each specific domain is described in eTable 2 in Supplement 1.

### Personalized Prehabilitation and Preoperative Cognitive Function, Physical Function, and Postoperative Complication Severity

**Figure 2.**
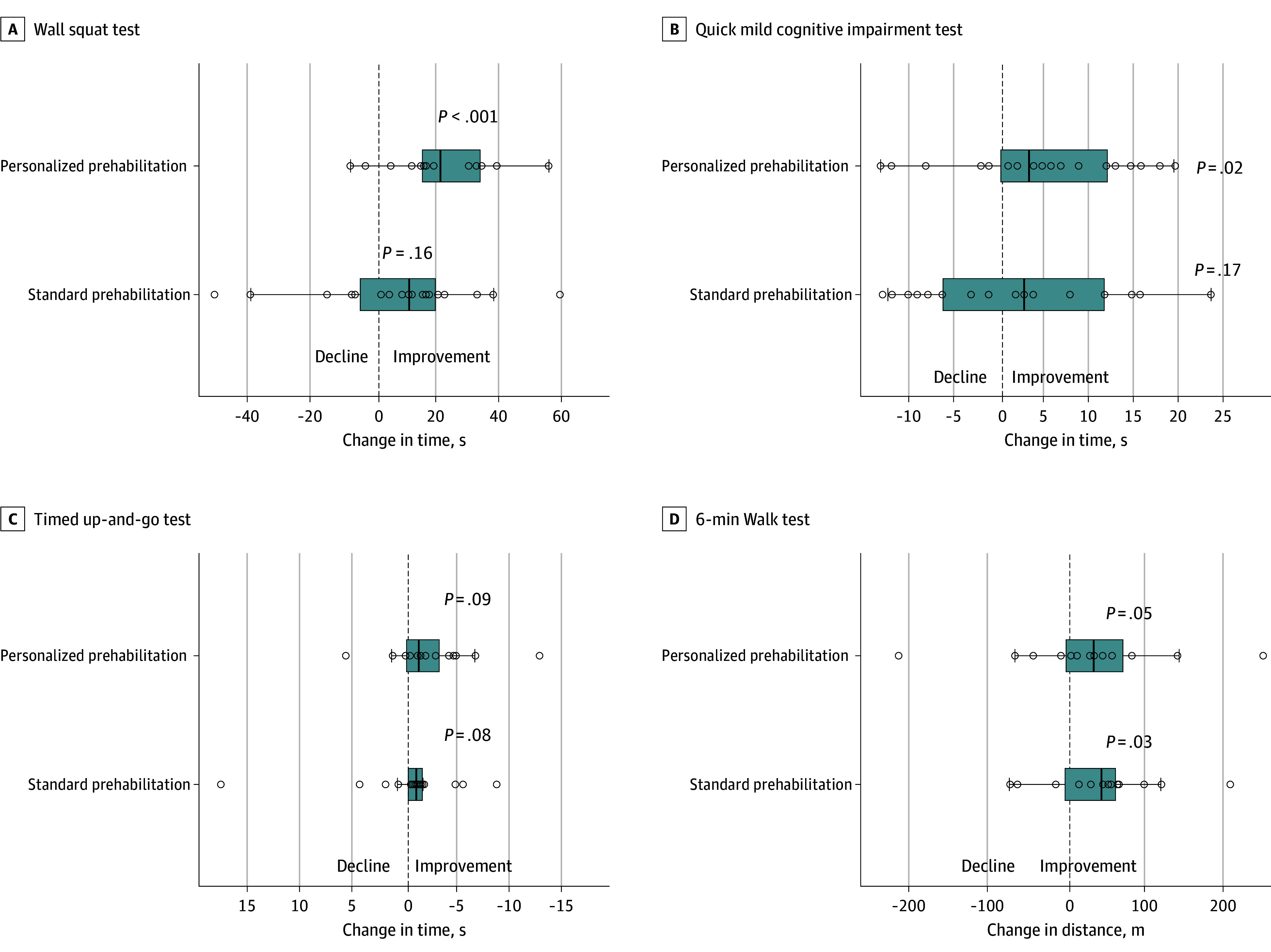
Physical and Cognitive Assessment Evolution After Prehabilitation Medians and IQRs are shown by the vertical bars and boxes, respectively; individual values are represented as dots. Whiskers show the upper and lower bounds.

Functional evaluation after prehabilitation revealed that patients in the personalized prehabilitation group significantly improved both their physical function (median [IQR] 6MWT in meters, 496 [340-619] before prehabilitation vs 546 [350-728] after prehabilitation, *P* = .05; median [IQR] timed up-and-go test in seconds, 9.0 [6.7-9.9] before prehabilitation vs 6.9 [5.3-8.5] after prehabilitation, *P* = .01; and median [IQR] wall squat time in seconds, 37.4 [26.3-59.2] before prehabilitation vs 66.2 [34.2-92.8] after prehabilitation, *P* < .001) as well as cognitive function (median [IQR] qMCI score, 66 [56-74] before prehabilitation vs 72 [65-76] after prehabilitation, *P* = .03). In contrast, patients from the standard prehabilitation group only improved in the 6MWT (median [IQR], 507 [368-623] before prehabilitation vs 573 [425-707] after prehabilitation, *P* = .03), with no significant gains in other metrics (Figure 2).

Significantly fewer patients in the personalized group experienced moderate to severe postoperative complications (Clavien-Dindo classification^34^ grade >1) compared to the standard prehabilitation group (4 vs 11, respectively; *P* = .04 [Mann-Whitney *U* test]) (eTable 3 in Supplement 1). No difference was observed in hospital length of stay in days (median [IQR], 2.1 [0.7-4.0] vs 2.6 [1.5-6.0], respectively; *P* = .48).

### Single-Cell Intracellular Signaling Activities for the Differentiation of Patients Before and After Prehabilitation

**Figure 3.**
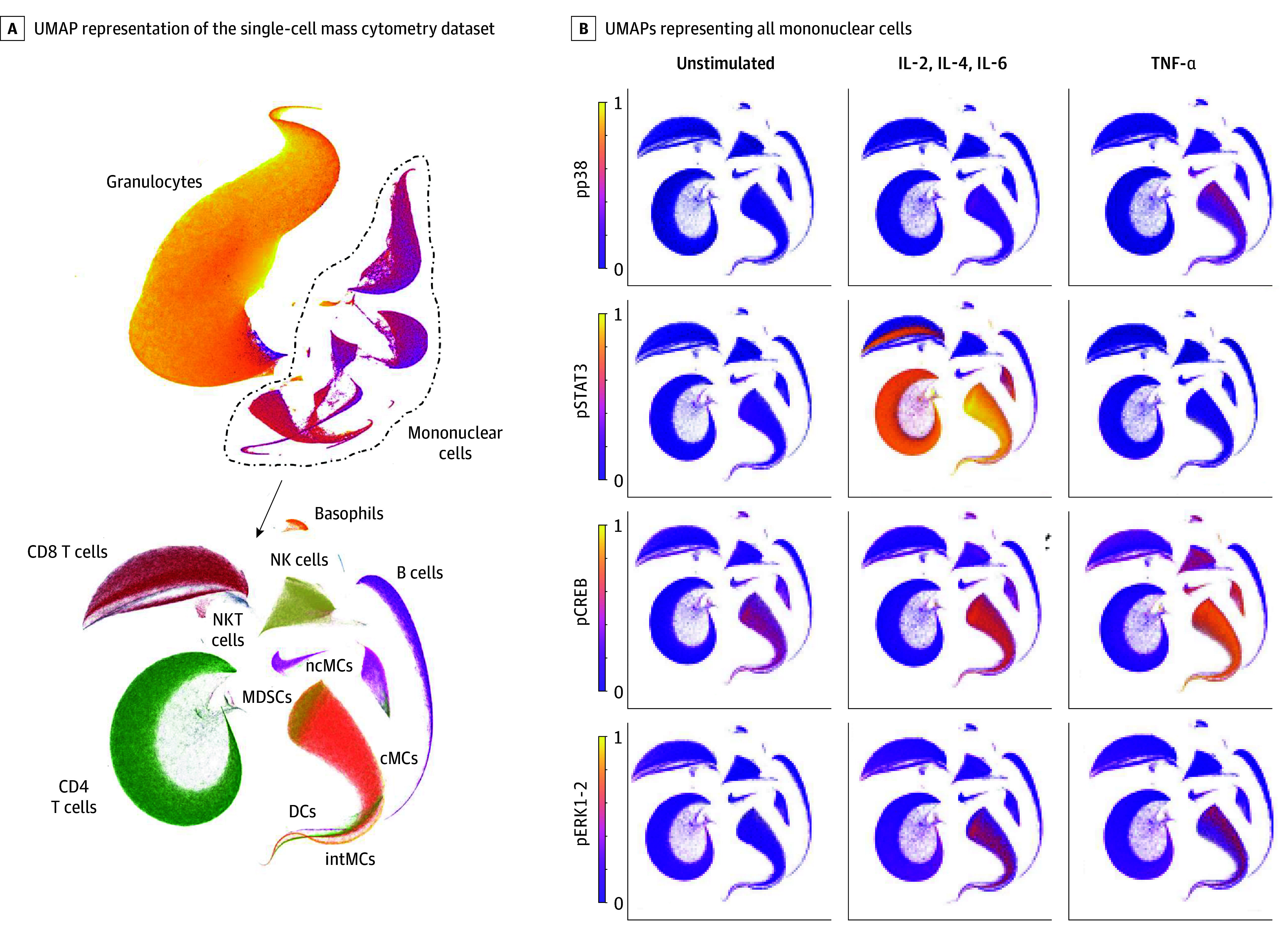
In Vitro Stress Test Before Surgery Analyzed With Mass Cytometry and Cell-Type– and Signaling-Specific Responses A, Uniform manifold approximation and projection (UMAP) representation of the single-cell mass cytometry dataset is shown. The upper panel includes all live leukocytes, including neutrophils and mononuclear cells; the lower panel, mononuclear cells only. UMAPS are clustered by cell type and annotated. B, UMAPs representing all mononuclear cells are colored according to levels of intracellular signaling in endogenous state (unstimulated) and after in vitro stimulation with interleukin (IL) 2, 4, and 6 and tumor necrosis factor (TNF-α). cMCs indicates classical monocytes; pCREB, phosphorylated cyclic adenosine monophosphate response–element binding protein; DCs, dendritic cells; ERK, endoplasmic reticulum kinase; intMCs, intermediate monocytes; pSTAT, phosphorylated signal transducer and activator of transcription; mDCs, myeloid dendritic cells; MDSCs, myeloid-derived suppressor cells; ncMCs, nonclassical monocytes; NK, natural killer; NKT, natural killer T cells; pDCs, plasmacytoid dendritic cells; pERK, phosphorylated kinase R-like endoplasmic reticulum kinase; pp, phosphoprotein.

To investigate whether prehabilitation modulates patients’ immunome, a 47*-*parameter mass cytometry assay was used to extract single-cell immune features from blood samples collected before and after prehabilitation. Uniform manifold approximation and projection analysis was performed to provide a visual synopsis of all single-cell immune features measured, including major innate and adaptive immune cell subset frequencies, their endogenous intracellular activities (eg, phosphorylation states), and capacities of each population to respond to a series of extracellular immune perturbations, including lipopolysaccharide, tumor necrosis factor (TNF) α, and a combination of interleukins (IL) 2, 4, and 6 (Figure 3). This approach provided a single-cell atlas of immune cell type-specific changes in intracellular signaling states in response to prehabilitation (eFigure 3 in Supplement 1).

Immune cell subsets were further evaluated using an established manual gating strategy, yielding a total of 1096 immune cell frequency and signaling features. The sparse machine learning algorithm Stabl^43^ was used to determine whether patients’ immunome differed after prehabilitation compared to baseline in each arm. The Stabl analysis identified a robust multivariable model that accurately differentiated samples collected before and after prehabilitation in the personalized prehabilitation group (AUROC, 0.88; 95% CI, 0.79-0.97; *P* < .001). In contrast, the multivariable analysis did not successfully differentiate samples before and after prehabilitation in the standard prehabilitation group (AUROC, 0.63; 95% CI, 0.48-0.78; *P* = .12). Between-group comparison of immune difference before and after prehabilitation did not yield a statistically significant model (AUROC, 0.58; 95% CI, 0.41-0.73; *P* = .34).

**Figure 4.**
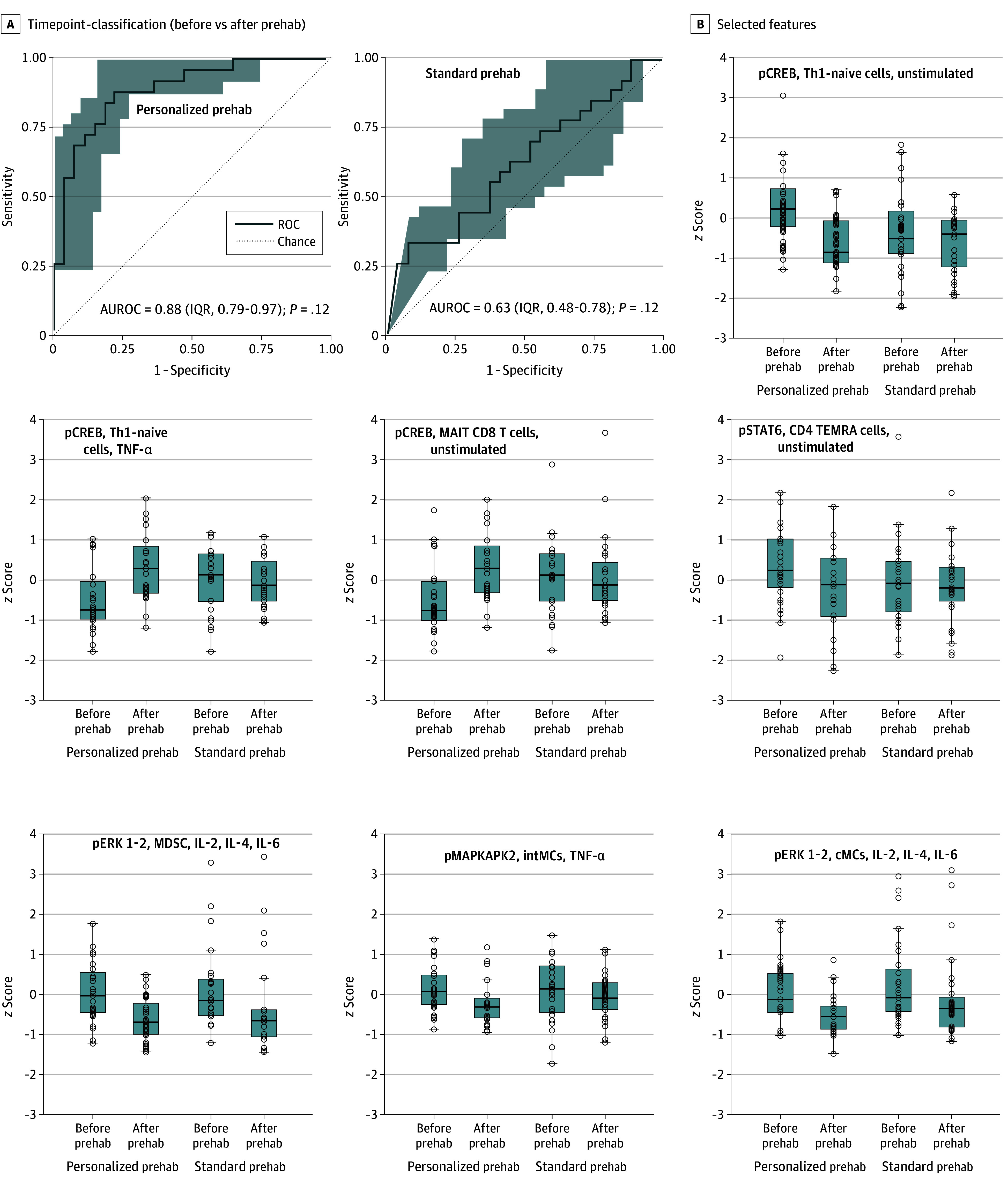
Multivariable Classification of Immune Profiles Before and After Prehabilitation A, Classification performance of the multivariable model for identifying immune modifications after prehabilitation in both groups. B, Medians and IQRs of the single-cell mass cytometry features selected by the model are shown by horizontal bars and boxes, respectively; individual values are represented as dots. Whiskers show the upper and lower bounds. AUROC indicates area under the ROC; cMCs, classical monocytes; IL, interleukin; intMCs, intermediate monocytes; MAIT, mucosal-associated invariant T cells; MAPKAPK2, mitogen-activated protein kinase-activated protein kinase 2; MDSCs, myeloid-derived suppressor cells; pCREB, phosphorylated cyclic adenosine monophosphate response–element binding protein; pERK, phosphorylated kinase R-like endoplasmic reticulum kinase; pMAPKAPK2, phosphorylated mitogen-activated protein kinase-activated protein kinase 2; prehab, prehabilitation; ROC, receiver operating characteristic curve; TEMRA, terminally differentiated effector memory T cells; Th1, T helper type 1; TNF-α, tumor necrosis factor α.

Analysis of features selected by the Stabl model revealed cellular elements of a patient’s immunome that were the most reliably altered by personalized prehabilitation (Figure 4; eFigure 3 in Supplement 1). In adaptive cell types, personalized prehabilitation resulted in decreased endogenous phosphorylated cyclic adenosine monophosphate response–element binding protein signaling in Th1-naive and mucosal-associated invariant CD8^+^ T cells, with increased reactivity to exogenous TNF-α stimulation, as well as a decreased endogenous phosphorylated signal transducer and activator of transcription-6 signaling in terminally differentiated effector memory CD4^+^ T cells. In innate immune populations, personalized prehabilitation decreased protein kinase R-like endoplasmic reticulum kinase signaling in response to IL-2, 4, and 6 in myeloid-derived suppressor cells and classical monocytes, as well as mitogen-activated protein kinase-activated protein kinase 2 signaling in response to TNF-α in intermediate monocytes.

These findings suggest that personalized, but not standard, prehabilitation exerted an immunomodulatory effect on the immunome. Specifically, the results show that personalized prehabilitation induces a dampening of basal Janus kinase/signal transducer and activator of transcription signaling in adaptive immunity and a decrease in myeloid differentiation primary response 88 (MyD88) reactivity to stimulation in innate cells indicative of a reduction of the basal inflammatory state after prehabilitation.

## Discussion

In this randomized clinical trial, we conducted an extensive analysis of the effects of standard vs personalized prehabilitation on patients’ immunological, physical, and cognitive function before major surgery. Our findings show that patients following a remotely delivered, personalized prehabilitation regimen had increased adherence, improved physical and cognitive function after prehabilitation, and fewer moderate to severe postoperative complications. Single-cell intracellular signaling analyses further revealed that personalized, but not standard, prehabilitation significantly modulated patients’ immunomes, with a dampening of basal Janus kinase/signal transducer and activator of transcription signaling and MyD88 signaling in adaptive immune cells, and a reduction of both basal and stimulated MyD88 pathway activity in innate immune cells, suggesting a reduction in proinflammatory pathways.

From a clinical standpoint, our results dovetail with previous studies examining the impact of prehabilitation on postoperative outcomes, showing that prehabilitation increases patients’ physiological reserve and improves recovery.^44^ Improvement in physical, cognitive, and postoperative outcomes were more pronounced in the personalized than the standard prehabilitation group, supporting the efficacy of providing coaching support tailored to each patient during their preoperative journey. Notably, the personalized prehabilitation program was delivered entirely through remote coaching of patients as our trial was conducted during the COVID-19 pandemic. As reported in other studies, patient adherence remained strong throughout the program.^45^ Previous studies have found that patients prefer remote administration over onsite prehabilitation^17^ as home-based programs minimize logistical barriers, reduce the burden on caretakers, and are more amenable, all of which likely contributed to our observed outcomes.^17,46^ Another key factor that may contribute to the superior efficacy of personalized prehabilitation over standard prehabilitation is the constant tailoring of the prehabilitation. Twice-weekly individual remote coaching with a physical therapist and a physician may facilitate the development of a realistic plan for progress in each domain, as well as an element of personal accountability. This approach placed patients in a positive feedback loop, helping them recognize their accomplishments and fostering motivation.^47^ The minimal effective mentoring dose needs to be further evaluated in subsequent studies to increase scalability and cost-effectiveness.

Meta-analyses^10,48^ on prehabilitation efficacy highlight the necessity of targeting high-risk patients and monitoring intervention effectiveness before surgery. Our findings support this perspective: the more resource-intensive, coached version of prehabilitation yielded significant physical, cognitive, and immunological improvements. These outcomes corroborate the need, and provide an avenue, for risk stratification and progress monitoring—for example, though a thorough biological and clinical assessment of frailty.

Notably, several key immune features modulated by personalized prehabilitation in our study resonate with features previously associated with surgical site infection^43,49^ and postoperative neurocognitive decline,^35^ suggesting they are interesting candidates for immune-based risk diagnostics and prehabilitation monitoring. For example, patients at risk of surgical site infection display an exacerbated MyD88 response after surgery,^43,49^ which was dampened by personalized prehabilitation, as we observed a reduction in mitogen-activated protein kinase-activated protein kinase 2 signaling in response to an exogenous stressor (TNF-α) in intermediate monocytes and protein kinase R-like endoplasmic reticulum kinase signaling in response to IL-2, 4, and 6 in classical monocytes and myeloid-derived suppressor cells. Similarly, increased phosphorylated cyclic adenosine monophosphate response–element binding protein response in adaptive cells following personalized prehabilitation may be clinically meaningful. Patients at risk for postoperative cognitive decline exhibit dampened phosphorylated cyclic adenosine monophosphate response–element binding protein response in adaptive cells to exogenous stress, this indicates that prehabilitation may mitigate postoperative cognitive decline risk after surgery.^35^ Interestingly, dampened MyD88 response to exogenous stress is a hallmark of immunosenescence,^50,51^ a dynamic and multifactorial process affecting both innate and adaptive immune cells. It also plays an important role in inflammaging and frailty seen in patients at risk of postoperative cognitive decline^35^ and other frailty-related postoperative complications.^52,53,54,55^ Our results show that prehabilitation helped to mitigate this ill-adapted immune response, corroborating other work^56,57,58,59,60^ that has shown an improvement in immune function with preoperative exercise and nutrition optimization. Our findings provide further insights on how adherence to a personalized prehabilitation program increases functional reserve, encompassing physical strength, cognitive abilities and immune health,^7,8,9,10,61^ allowing patients to increase their resilience and better adapt to surgical stress.

### Limitations

This study has several limitations. All patients underwent a prehabilitation regimen, limiting our ability to compare its effect to no intervention. Each patient therefore served as their own control. The heterogeneity of surgical procedures between groups may have introduced variability in postoperative outcomes. This variability should be mitigated by the randomized design of the study, providing robust foundation for our findings. Stratification on surgical procedures should be considered in future studies with higher sample size. While mass cytometry allows for simultaneous detection of up to 50 parameters at a single-cell level, the technology necessitates preselection of cell-surface and intracellular features. Future studies, including untargeted transcriptomic, proteomic, or metabolomic approaches, are required to provide a more comprehensive profile of patients’ inflammatory, metabolic, and hormonal responses to prehabilitation. Despite these limitations, our study finds strong evidence of immune and clinical changes after personalized prehabilitation, warranting subsequent, more powered trials to validate our findings.

## Conclusions

In conclusion, the findings in this study demonstrate a significant effect of a personalized prehabilitation regimen on physical, cognitive, and immune function. The study identifies key features echoing previous work looking at the prediction of surgical site infection and postoperative neurocognitive decline after surgery^35,43,49^ that are positively modulated with prehabilitation. Identification of cell-specific immune features reliably modulated in the personalized prehabilitation group provides a promising set of immune biomarkers that may form the foundation for immune-based diagnostics to identify high-risk patients most likely to benefit from personalized prehabilitation. Moreover, these biomarkers may serve as surrogate end points to evaluate preoperative readiness and their risk for postoperative complications throughout their preoperative journey. Tailoring prehabilitation programs to individual patients based on objective immunological end points will be crucial for the development of data-driven and effective prehabilitation interventions for high-risk surgical patients.

## Notes

Supplement 1. Trial protocol

Supplement 2. eMethods eFigure 1. Immune cell gating strategy eFigure 2. Study design and workflow eFigure 3. Immune distribution and signaling response before and after prehab eTable 1. Mass Cytometry antibody panel eTable 2. adherence score per specific prehab domain eTable 3. Postoperative outcomes eTable 4. Evolution of major cell type frequencies after prehab

Supplement 3. Data sharing statement
