# Supplementary material for: Immune Modulation by Personalized vs Standard Prehabilitation Before Major Surgery: A Randomized Clinical Trial
Source: JAMA Surg. 2025 Nov 12;161(1):20–30. doi: 10.1001/jamasurg.2025.4917 (PMC12613092; doi:10.1001/jamasurg.2025.4917)
Supplement: Supplement 1. — Trial protocol [file jamasurg-e254917-s001.pdf]

PROTOCOL
APPLICATION FORM
Human Subjects Research
Stanford University

Title : Immune system modulation by enhanced vs standard prehabilitation program in patients undergoing elective major surgery - a prospective monocentric randomized single-blinded controlled trial.

Approval Period: 10/23/2023 - 12/31/2999

Personnel Info 3
Participant Population 4
Study Location 4
General Checklist 5
Funding 7
Resources 7
Expedited Category 8
Purpose 9
Radioisotopes or Radiation Machines 14
Drugs, Reagents, Chemicals, Devices 15
Medical Equipment for Human Subjects and Laboratory Animals 15
Participant Population(a-g) 15
Participant Population(h-m) 17
Risks(a-d) 17
Privacy And Confidentiality 19
Conflict Of Interest 20
Consent Background 21
Assent Background 22
Hipaa 22
Attachments 22

Obligations 23

Title : Immune system modulation by enhanced vs standard prehabilitation program in patients undergoing elective major surgery - a prospective monocentric randomized single-blinded control . . .

Approval Period: 10/23/2023 - 12/31/2999

Protocol Director

Name		Degree (Program/year if student)		Position, e.g. Assistant Professor, Resident, etc.
Cindy Kin		MD		Asst Prof-Med Ctr Line
**Department** Surgery - General Surgery	5641	**Phone** 650-646-0014	null	**E-mail** cindykin@stanford.edu
**CITI Training current**				Y

Admin Contact

Name		Degree (Program/year if student)		Position, e.g. Assistant Professor, Resident, etc.
Martha Sample Tingle		RN		Research Nurse Manager
**Department** Anesthesia	5117	**Phone** (650) 724-2742	(650) 725-8052	**E-mail** mtingle@stanford.edu
**CITI Training current**				Y

Investigator

Name		Degree (Program/year if student)		Position, e.g. Assistant Professor, Resident, etc.
Brice Louis Jules Gaudilliere		M.D., Ph.D.		Asst Prof-Med Ctr Line
**Department** Anesthesia	5640	**Phone** +1 (617) 230-5927#	null	**E-mail** gbrice@stanford.edu
**CITI Training current**				Y

Other Contact

Name		Degree (Program/year if student)		Position, e.g. Assistant Professor, Resident, etc.
Franck Verdonk		MD PhD		Postdoctoral fellow
**Department** Anesthesiology, Perioperative and Pain Medicine	5640	**Phone** 6504416912	null	**E-mail** fverdonk@stanford.edu
**CITI Training current**				Y

Academic Sponsor

Name		Degree (Program/year if student)		Position, e.g. Assistant Professor, Resident, etc.
**Department**		**Phone**		**E-mail**
**CITI Training current**				

Other Personnel

Name	Degree (Program/year if	Position, e.g. Assistant Professor,

Title : Immune system modulation by enhanced vs standard prehabilitation program in patients undergoing elective major surgery - a prospective monocentric randomized single-blinded control . . .

Approval Period: 10/23/2023 - 12/31/2999

Dyani Kalea Gaudilliere		**student)**		**Resident, etc.**<br>Clinical Assistant Professor
**Department**		**Phone**<br>(650) 725-6946		**E-mail**<br>dyani00@stanford.edu
**CITI Training current**				Y

**Name**<br>Amy Sung Tsai		**Degree (Program/year if student)**		**Position, e.g. Assistant Professor, Resident, etc.**<br>Casual - Non-Exempt
**Department**<br>Anesthesia - Adult Pain (Designated)	5117	**Phone**		**E-mail**<br>astsai@stanford.edu
**CITI Training current**				Y

Participant Population(s) Checklist — Yes/No

- Children (under 18) N
- Pregnant Women and Fetuses N
- Neonates (0 - 28 days) N
- Abortuses N
- Prisoners N
- International Participants N
  Please enter the countries separated by comma
- Impaired Decision Making Capacity N
- Cancer Subjects N
- Laboratory Personnel N
- Healthy Volunteers N
- Students N
  Stanford students N Other students N
- Employees N
- Other (i.e., any population that is not specified above) Y

Study Location(s) Checklist — Yes/No

- Stanford University Y
- Clinical & Translational Research Unit (CTRU)
- Stanford Medicine Health Care Y
  Tri-Valley
- Stanford Medicine Children's Health
- VAPAHCS (Specify PI at VA)
- Other (Click ADD to specify details)

Title : Immune system modulation by enhanced vs standard prehabilitation program in patients undergoing elective major surgery - a prospective monocentric randomized single-blinded control . . .

Approval Period: 10/23/2023 - 12/31/2999

General Checklist

**Multi-site**	**Yes/No**
• Is this a multi-site study? A multi-site study uses the same protocol to conduct human subjects research at more than one site.	N

**Cooperative/Collaborative Study?**	**Yes/No**
• Are there any collaborating institution(s)? A collaborating institution is generally an institution that collaborates equally on a research endeavor with one or more institutions.	N

**Cancer Institute**	**Yes/No**
• Cancer-Related Studies (studies with cancer endpoints), Cancer Subjects (e.g., clinical trials, behavior/prevention) or Cancer Specimens (e.g., blood, tissue, cells, body fluids with a scientific hypothesis stated in the protocol).	N

**Clinical Trials**	**Yes/No**
• Investigational drugs, biologics, reagents, or chemicals?	N
• Commercially available drugs, reagents, or other chemicals administered to subjects that are being studied?	N
• Investigational Medical Device / Commercial Medical Device used off-label or if being studied?	N
• IDE Exempt Device (Commercial Medical Device used according to label, Investigational In Vitro Device or Assay, or Consumer Preference/Modifications/Combinations of Approved Medical Devices)	N
• Will this study be registered on clinicaltrials.gov? (See Stanford decision tree)	Y
• Who will register for ClinicalTrials.gov?<br>NCT# 04498208	Y

**Tissues and Specimens**	**Yes/No**
• Human blood, cells, tissues, or body fluids (tissues)?	Y
• Tissues to be stored for future research projects?	N
• Tissues to be sent out of this institution as part of a research agreement? For guidelines, please see Material Transfer Agreements	N

**Biosafety (APB)**	**Yes/No**
• Are you submitting a Human Gene Transfer investigation using a biological agent or	N

recombinant DNA vector? If yes, please complete the Gene Transfer Protocol Application Supplemental Questions and upload in Attachments section.

- Are you submitting a Human study using biohazardous/infectious agents? If yes, refer to the https://ehs.stanford.edu/forms-tools/genome-editing-and-gene-drives-stanford Administrative Panel on BioSafety website prior to performing studies. — N
- Are you submitting a Human study using samples from subjects that are known or likely to contain biohazardous/infectious agents? If yes, refer to the https://ehs.stanford.edu/forms-tools/genome-editing-and-gene-drives-stanford Administrative Panel on BioSafety website prior to performing studies. — N

**Human Embryos or Stem Cells**	**Yes/No**
• Human Embryos or Gametes?	N
• Human Stem Cells (including hESC, iPSC, cancer stem cells, progenitor cells)	N

**Veterans Affairs (VA)**	**Yes/No**
• The research recruits participants at the Veterans Affairs Palo Alto Health Care System(VAPAHCS).	N
• The research involves the use of VAPAHCS non-public information to identify or contact human research participants or prospective subjects or to use such data for research purposes.	N
• The research is sponsored (i.e., funded) by VAPAHCS.	N
• The research is conducted by or under the direction of any employee or agent of VAPAHCS (full-time, part-time, intermittent, consultant, without compensation (WOC), on-station fee-basis, on-station contract, or on-station sharing agreement basis) in connection with her/his VAPAHCS responsibilities.	N
• The research is conducted using any property or facility of VAPAHCS.	N

**Equipment**	**Yes/No**
• Use of Patient related equipment? If Yes, equipment must meet the standards established by Biomedical Engineering (BME) (650-725-5000)	N
• Medical equipment used for human patients/subjects also used on animals?	N
• Radioisotopes/radiation-producing machines, even if standard of care? ; More Info	N

**Payment**	**Yes/No**
• Subjects will be paid/reimbursed for participation? See payment considerations.	N

**Funding**	**Yes/No**
• Training Grant?	N

- Program Project Grant? N
- Federally Sponsored Project? N
- Industry Sponsored Clinical Trial? N

Funding

Funding - Grants/Contracts/Agreements

Funding - Fellowships

Gift Funding

Dept. Funding

Department Name : Anesthesiology and Perioperative Medicine

Other Funding

Other Fund Name : Cindy Kin faculty start-up funds

Resources :

a) Qualified staff.

Please state and justify the number and qualifications of your study staff.

> Cindy Kin, MD, colorectal surgeon at Stanford, PD of study - responsible for protocol development

> Brice Gaudilliere, MD PhD, PI of study,
- responsible for enrolling subjects, data collection, data analysis, interpretation, and overseeing all aspects of the study

> Dyani Gaudilliere, Franck Verdonk, Martha Tingle - data collection, data analysis, interpretation, protocol development, coaching

> Sydni Au Hoy - data management, OnCore updates

b) Training.

Describe the training you will provide to ensure that all persons assisting with the research are informed about the protocol and their research-related duties and functions.

The entire team will have an in-person or phone/teleconference meeting weekly to discuss the protocol, enrollment issues, data collection issues, and data analysis and interpretation.

c) Facilities.

Provide the location(s) where the research will be conducted, including physical address if not conducted on site at Stanford University, Stanford Hospital on Pasteur Dr., Lucile Packard Children's Hospital on Welch Rd. or VAPAHCS. Describe the facilities and resources available to conduct the research at these sites.

Stanford Health Care: surgical patients will undergo study enrollment and education in the outpatient general surgery clinics
Stanford Department of Surgery: This is where the data analysis and interpretation will occur.

d) Sufficient time.

Explain the time that you and your research team will allocate to perform the research activities, including data analysis.

Our goal enrollment is 120 patients. We anticipate that we will enroll 6 patients a month, so we estimate that we will enroll patients for 13 months and follow them for 3 months (Total 16 months).

e) Access to target population.

Explain and justify whether you will have access to a population that will allow recruitment of the required number of participants.

As the PD is a surgeon who works closely with other surgeons in multiple departments, we have direct access to the surgery patient population. Our research workflow is well-embedded into the clinical workflow as we have been enrolling patients in these clinics since 2018.

f) Access to resources if needed as a consequence of the research.

State whether you have medical or psychological resources available that participants might require as a consequence of the research when applicable. Please describe these resources.

We do not anticipate any medical or psychological consequences as a result of the study. However, as the PI is a practicing surgeon, we have access to medical or psychological resources that participants might require.

g) Lead Investigator or Coordinating Institution in Multi-site Study.

Please explain (i) your role in coordinating the studies, (ii) procedures for routine communication with other sites, (iii) documentation of routine communications with other sites, (iv) planned management of communication of adverse outcomes, unexpected problems involving risk to participants or others, protocol modifications or interim findings.

Expedited Form

A protocol must be no more than minimal risk (i.e., "not greater than those ordinarily encountered in daily life") AND must only involve human subjects in one or more of the following paragraphs.

Select one or more of the following paragraphs:

1. N **Clinical studies of drugs and medical devices only when condition (a) or (b) is met.**
   a) Research on drugs for which an investigational new drug application (21 CFR Part 312) is not required. (Note: Research on marketed drugs that significantly increases the risks or decreases the acceptability of the risks associated with the use of the product is not eligible for expedited review.)
   b) Research on medical devices for which
      i) an investigational device exemption application (21 CFR Part 812) is not required; or

ii) the medical device is cleared/approved for marketing and the medical device is being used in accordance with its cleared/approved labeling.

2. N **Collection of blood samples by finger stick, heel stick, ear stick, or venipuncture as follows:**
   a) from healthy, nonpregnant adults who weigh at least 110 pounds. For these subjects, the amounts drawn may not exceed 550 ml in an 8 week period and collection may not occur more frequently than 2 times per week; or
   b) from other adults and children, considering the age, weight, and health of the subjects, the collection procedure, the amount of blood to be collected, and the frequency with which it will be collected. For these subjects, the amount drawn may not exceed the lesser of 50 ml or 3 ml per kg in an 8 week period and collection may not occur more frequently than 2 times per week.

3. N **Prospective collection of biological specimens for research purposes by non invasive means.**

4. Y **Collection of data through non invasive procedures (not involving general anesthesia or sedation) routinely employed in clinical practice, excluding procedures involving x-rays or microwaves. Where medical devices are employed, they must be cleared/approved for marketing. (Studies intended to evaluate the safety and effectiveness of the medical device are not generally eligible for expedited review, including studies of cleared medical devices for new indications.)**
   Examples:
   a) physical sensors that are applied either to the surface of the body or at a distance and do not involve input of significant amounts of energy into the subject or an invasion of the subject's privacy;
   b) weighing or testing sensory acuity;
   c) magnetic resonance imaging;
   d) electrocardiography, electroencephalography, thermography, detection of naturally occurring radioactivity, electroretinography, ultrasound, diagnostic infrared imaging, doppler blood flow, and echocardiography;
   e) moderate exercise, muscular strength testing, body composition assessment, and flexibility testing where appropriate given the age, weight, and health of the individual.

5. N **Research involving materials (data, documents, records, or specimens) that have been collected, or will be collected solely for nonresearch purposes (such as medical treatment or diagnosis). (NOTE: Some research in this paragraph may be exempt from the HHS regulations for the protection of human subjects. 45 CFR 46.101(b)(4). This listing refers only to research that is not exempt.)**

6. N **Collection of data from voice, video, digital, or image recordings made for research purposes.**

7. Y **Research on individual or group characteristics or behavior(including, but not limited to, research on perception, cognition, motivation, identity, language, communication, cultural beliefs or practices, and social behavior) or research employing survey, interview, oral history, focus group, program evaluation, human factors evaluation, or quality assurance methodologies. (NOTE: Some research in this category may be exempt from the HHS regulations for the protection of human subjects. 45 CFR 46.101(b)(2) and (b)(3). This listing refers only to research that is not exempt.)**

1. Purpose

a) In layperson's language state the purpose of the study in 3-5 sentences.

Over 30 million surgeries are performed annually in the US. Up to 30% of surgical patients experience delayed surgical recovery, marked by prolonged post-surgical pain, opioid consumption, and functional

impairment, which contributes $8 billion annually to US health care costs. Novel interventions that improve the resolution of pain, minimize opioid exposure, and accelerate functional recovery after surgery are urgently needed.

Multi-modal pre-operative optimization programs (or "prehab") integrating exercise, nutrition, and stress reduction have been shown to safely and effectively improve outcomes after surgery. However, we lack objective biological markers to assess prehab effectiveness and to tailor prehab programs to individual patients. Surgery is a profound immunological perturbation, during which a complex network of innate and adaptive immune cells is mobilized to organize the recovery process of wound healing, tissue repair, and pain resolution. As such, the in-depth assessment of a patient's immune system before surgery is a highly promising approach to identify biological markers for accurate risk prediction.

b) State what the Investigator(s) hope to learn from the study. Include an assessment of the importance of this new knowledge.

The investigators hope to determine whether a personalized health optimization program combining one-on-one coaching, tailored to each patient ("surge") is able to enhance patient's immune state in comparison to unpersonnalized prehabilitation ("standard rehabilitation") before surgery

The importance of this new knowledge is better understanding of ways that we can improve outcomes, engagement, experience and overall long-term health of patients undergoing major abdominal operations. This would translate to increased healthcare value and better long-term outcomes.

c) Explain why human subjects must be used for this project. (i.e. purpose of study is to test efficacy of investigational device in individuals with specific condition; purpose of study is to examine specific behavioral traits in humans in classroom or other environment)

The purpose of the study is to determine the effect on immune cells of a personalized prehabilitation intervention in adults undergoing elective major abdominal surgery.

2. Study Procedures

a) Please SUMMARIZE the research procedures, screening through closeout, which the research participant will undergo. Sections in the protocol attached in section 16 can be referenced, BUT do not copy the clinical protocol. Be clear on what is to be done for research and what is part of standard of care. For research involving collaborators, please specify the respective roles of Stanford and each collaborator on the protocol.

Standard prehabilitation:
Patients will be provided with standard instructions in a hard-copy form specific to prehabilitation before surgery associating physical, nutritional, stress-reduction and cognitive recommendations without any personalized coaching for at least 3 weeks prior to surgery (see attached ControlGroup_ProgramBook document).

Research Activities:

1. Screening: The research team will identify eligible patients by screening the clinic schedules of general surgeons.

2. Study Enrollment: Either in person during their surgical clinic visit, or by phone at least 28 days before surgery. If patients accept to be included, they will be randomized.

3. Baseline assessment (Day 0): Nutritional (using the Mediterranean Diet Scale), physical (by measuring 6 min walk test, Timed up and go, Five Times Sit to Stand Test and Wall Squat Tests), cognitive (using the qMCI test), anxiety (using the APAIS scale and the Pain Catastrophizing Scale) and quality of life status (using the SF36-scale) at baseline (prior to prehabilitation) will be recorded on RedCap by a personnel previously trained to the achievement of tests of the study.

4. Prehabilitation: SURGE or standard program will be proposed to the patients for 3-6 weeks.

SURGE consists in a 6-session (over 3-6 weeks) personalized prehabilitation program. This progam will be delivered remotely with 2 in person (with a trained member of the research team) sessions every week. Self-exercises will be promoted every day.

>> Exercise Component: participants will have the opportunity to make a personalized plan with a member of the research team (SURGE) for physical exercise. First, an evaluation of patient's aerobic capacity, functional mobility, functional strength, ambulation, fall risk, and overall mobility using validated outcome measures and movement analysis will be done by a trained member of the research team. Based on their functional level and preexisting conditions, therapeutic exercises will be recommended to optimize their strength, endurance, flexibility, and functional mobility in preparation for surgery. Therapeutic exercises may include gait training, strengthening exercises, flexibility exercises/stretches, movement pattern training, relaxation techniques, neuromuscular re-education, breathing strategies, movement strategies, and transfer training (i.e. bed mobility). Decisions about the interventions are based on the assessment, individual patient characteristics, environmental factors (i.e. space limitations), surgical type, and monitoring of the client's response and progress. Weekly sessions are 30-45 minutes long to monitor the client's response and progress their program. Based on their response to exercise (evaluated also by one app: Timed Walk App), re-evaluation via the assessments described above, change their plan of care (alter frequency, resistance, exercises) or discontinuation of treatment could be decided.

>> Nutrition Component: participants will have the opportunity to make a personalized plan for improving their diet with a member of the research team (SURGE). It will involve transitioning the patient to the Mediterranean diet with targets regarding hydration and the proportion of fruits, vegetables, whole grains, and healthy fats the patient will consume in a given day. This nutritional program is flexible, and it allows for specific physician and nutritionist-recommended dietary needs to be incorporated. The patient is also given tools to encourage success in healthier eating, including recipes, sample menus, healthy restaurant options, meal kit options, and local CSA box options.

>> Cognition component: participants will have the opportunity to make a personalized plan for improving their cognition with a member of the research team (SURGE). It will consist on the daily use of Lumosity training program (https://www.lumosity.com/), an already existing free of charge app built to increase memory skills and used in the perioperative context. The program proposed by Lumosity is tailored to each patient. The use of Lumosity will be monitored at each in person session. Patients will be expected to complete at least three 5 minute sessions of Lumosity training per day. No information will be collected by the company.

>> Stress-reduction component: participants will have the opportunity to learn techniques for mindfulness that have been established to reduce stress and assist with pain management with a member of the research team (SURGE). Patients will be offered to listen to audio recording of relaxation sessions. Each session contains a relaxation technique that the patient practices while listening to the recording. Then the technique is used the next as a baseline to introduce a new technique more advanced. For some patients who struggle with the techniques the program will be adapted. Patients will choose with 2 or 3 techniques and work on them.

5. Every 7 days, whatever the arm, a compliance questionnaire will be filled by phone, by trained members of the research team blinded to the treatment arm of the study and collected in RedCap

6. End of prehabilitation program: Nutritional (using the Mediterranean Diet Scale), physical (by measuring 6 min walk test, Timed up and go, Five Times Sit to Stand Test and Wall Squat Test), cognitive (using the qMCI test), anxiety (using the APAIS scale and the Pain Catastrophizing Scale) and quality of life status (using the SF36-scale) at the end of the prehabiliation program will be recorded on RedCap by a personnel previously trained to the achievement of tests of the study.

7. Surgery

8. Post-operative period:
>> From surgery end to discharge from the hospital, length of stay, pain and adverse clinical events will be measured and recorded on RedCap.
>> Cognitive function (using qMCI), neuropathic pain (using the DN-4 scale) and quality of life (using SF-36 scale) will be assessed 30 days after surgery and recorded on RedCap.

b) Explain how the above research procedures are the least risky that can be performed consistent with sound research design.

These research procedures pose minimal risk to the patient, as the exercises are tailored for patients' baseline level of fitness, nutritional, cognitive and anxiety status to reduce the risk of injury. Moreover prehabilitation has been demonstrated to be safe and beneficial to surgical outcomes.

c) State if deception will be used. If so, provide the rationale and describe debriefing procedures. Since you will not be fully informing the participant in your consent process and form, complete an alteration of consent (in section 13). Submit a debriefing script (in section 16).

Deception will not be used.

d) State if photo, audio or video recording will occur. Describe what will become of the photos or recording after use, e.g., shown at scientific meetings, erased. Describe the final disposition of the recordings.

Audio/video recording will not occur.

e) Describe alternative procedures or courses of treatment, if any, that might be advantageous to the participant. Describe potential risks and benefits associated with these. Any standard treatment that is being withheld must be disclosed in the consent process and form. (i.e. standard-of-care drug, different interventional procedure, no procedure or treatment, palliative care, other research studies).

There are no proven alternative procedures for Prehab. Standard treatment is not being withheld.

f) Will it be possible to continue the more (most) appropriate therapy for the participant(s) after the conclusion of the study?

Patients may choose to continue any/all of the prehab program components as long as they wish.

g) Study Endpoint. What are the guidelines or end points by which you can evaluate the different treatments (i.e. study drug, device, procedure) during the study? If one proves to be clearly more effective than another (or others) during the course of a study, will the study be terminated before the projected total participant population has been enrolled? When will the study end if no important differences are detected?

The endpoints we will use to evaluate the adherence to prehab programs (compared to one arm to the other) will compare before and after prehab program time and are:

> Adherence to prehab programs using a modified "Rehabilitation Adherence Measure for Athletic Training" scale
> Physical status assessment including 6 Minute Walk Test (6MWT), Timed Up and Go (TUG), Five Times Sit to Stand Test & Wall Squat Test and Body Mass Index (BMI)
> Prevalence and global severity of anxiety using questionnaires such as the APAIS (Amsterdam Preoperative Anxiety and Information Scale) and the Pain catastrophization scale.
> Cognitive status evaluated by the qMCI

We will also evaluate the effect of prehab programs on post-operative outcomes by collecting:

> Hospital length of stay
> Readmission within 30 days after surgery
> Postoperative complications scored by the Comprehensive Complication Index (CCI) within 90 days after surgery
> Inpatient opioid use and pain levels during hospitalization
> Neuropathic pain, quality of life and cognitive function assessed 30 days after surgery (in comparison to before surgery)

If the intervention is found to be clearly harmful then the study will be terminated before the total projected participant population has been enrolled. This will be determined by assessing for increase in complication rates after surgery beyond our baseline rates of complications (as determined by NSQIP data). These rates will be

Title : Immune system modulation by enhanced vs standard prehabilitation program in patients undergoing elective major surgery - a prospective monocentric randomized single-blinded control . . .

Approval Period: 10/23/2023 - 12/31/2999

calculated on a quarterly basis.

The study will end after complete enrollment (120 patients) or after 16 months of enrollment into the prehab intervention if no important differences are detected.

3. Background

a) Describe past experimental and/or clinical findings leading to the formulation of the study.

A randomized controlled pilot study here at Stanford for colorectal surgery patients demonstrated better physical function and lower pain scores at 30 days after surgery in case of prehabilitation program.

However, prehab programs are still not common in practice. Major impediments include the lack of patient compliance.

So, we would like to compare compliance of personalized health optimization program with one-on-one coaching, tailored to each patient's predicted surgical recovery (SURGE program) vs.
a less-intensive approach.

b) Describe any animal experimentation and findings leading to the formulation of the study.

none.

4. Radioisotopes or Radiation Machines

a) List all standard of care procedures using ionizing radiation (radiation dose received by a subject that is considered part of their normal medical care). List all research procedures using ionizing radiation (procedures performed due to participation in this study that is not considered part of their normal medical care). List each potential procedure in the sequence that it would normally occur during the entire study. More Info

Identify Week/Month of study	Name of Exam	Identify if SOC or Research

b) For research radioisotope projects, provide the following radiation-related information:

Identify the radionuclide(s) and chemical form(s).

For the typical subject, provide the total number of times the radioisotope and activity will be administered (mCi) and the route of administration.

If not FDA approved provide dosimetry information and reference the source documents (package insert, MIRD calculation, peer reviewed literature).

c) For research radiation machine projects, provide the following diagnostic procedures:

For well-established radiographic procedures describe the exam.

For the typical subject, identify the total number of times each will be performed on a single research subject.

For each radiographic procedure, provide the setup and technique sufficient to permit research subject dose modeling. The chief technologist can usually provide this information.

For radiographic procedures not well-established, provide FDA status of the machine, and information sufficient to permit research subject dose modeling.

d) **For research radiation machine projects, provide the following therapeutic procedures:**

For a well-established therapeutic procedure, identify the area treated, dose per fraction and number of fractions. State whether the therapeutic procedure is being performed as a normal part of clinical management for the research participants's medical condition or whether it is being performed because the research participant is participating in this project.

For a therapeutic procedure that is not well-established, provide FDA status of the machine, basis for dosimetry, area treated, dose per fraction and number of fractions.

5. Devices

a) **Please list in the table below all Investigational Devices (including Commercial Devices used off-label) if they are being studied.**

b) **Please list in the table below all IDE Exempt Devices (Commercial Device used according to label, Investigational In Vitro Device or Assay, or Consumer Preference/Modifications/Combinations of Approved Devices) to be used on participants.**

6. Drugs, Reagents, or Chemicals and Devices

a) **Please list in the table below all investigational drugs, reagents or chemicals if they are being studied.**

b) **Please list in the table below all commercial drugs, reagents or chemicals if they are being studied.**

7. Medical Equipment for Human Subjects and Laboratory Animals

If medical equipment used for human patients/participants is also used on animals, describe such equipment and disinfection procedures.

none

8. Participant Population

a) **State the following: (i) the number of participants expected to be enrolled at Stanford-affiliated site(s); (ii)the total number of participants expected to enroll at all sites; (iii) the type of participants (i.e. students, patients with certain cancer, patients with certain cardiac condition) and the reasons for using such participants.**

(i) 120 patient participants total from Stanford Health Care. 15 surgeons from SHC.
(ii) total = 120 participants (only one site included in this study). 15 surgeons from SHC
(iii) participants will be patients undergoing elective major surgery. Reasons for using this group of patients is that they have a baseline significant risk of post-operative complications including surgical site infections

and readmissions. Finally, patients undergoing major abdominal surgery often feel overwhelmed, or a sense of loss of control over their bodies; therefore, we hope that this intervention would help support them and also give them a sense of agency in their own recovery and health.

b) State the age range, gender, and ethnic background of the participant population being recruited.

Age range: 18 or older
Gender: representative of the patient population of Stanford Health Care
Ethnic background: representative of the patient population of Stanford Health Care

c) State the number and rationale for involvement of potentially vulnerable subjects in the study (including children, pregnant women, economically and educationally disadvantaged, decisionally impaired, homeless people, employees and students). Specify the measures being taken to minimize the risks and the chance of harm to the potentially vulnerable subjects and the additional safeguards that have been included in the protocol to protect their rights and welfare.

Economically disadvantaged people will be able to take part in the study as it will not cost them anything to participate.
For educationally disadvantaged people who wish to take part in the program, we will determine which aspects of the program they would be able to do, based on their ability level. We will also ask patients' caregivers if they are able to help the patients with the program.
Decisionally impaired patients will not be included in the study.
Employees and students will be given the opportunity to participate, but will be explicitly assured that their decision to participate or not participate will have no bearing on their professional or educational status.
Children and pregnant women will not be enrolled.

d) If women, minorities, non-English speaking individuals, or children are not included, a clear compelling rationale must be provided (e.g., disease does not occur in children, drug or device would interfere with normal growth and development, etc.).

Children will not be included as children typically do not undergo these types of abdominal operations, and this study is being performed only in the adult surgery clinic.

e) State the number, if any, of participants who are laboratory personnel, employees, and/or students. They should render the same written informed consent. If payment is allowed, they should also receive it. Please see Stanford University policy.

If any eligible patients happen to also be laboratory personnel, employees, and/or students, and are interested in the study, then they may be included. They will go through the same RedCap informed consent process as other patients. Payment will not be given for these patients.

f) State the number, if any, of participants who are healthy volunteers. Provide rationale for the inclusion of healthy volunteers in this study. Specify any risks to which participants may possibly be exposed. Specify the measures being taken to minimize the risks and the chance of harm to the volunteers and the additional safeguards that have been included in the protocol to protect their rights and welfare.

None of the participants are healthy volunteers.

g) Describe your plan to identify and recruit potential participants including who will inform them about the study and how they will be initially contacted by the researchers (e.g., https://med.stanford.edu/spectrum/researcher-resources/participant-engagement/engagement-consultations.html Participant Engagement services; chart review; treating physician; ads including social media posts). All final or revised recruitment materials must be approved by the IRB before use. Contacting potential participants is not permitted prior to IRB approval. See https://stanfordmedicine.box.com/shared/static/8uebsdjrrqjyauanjp9i9d0gm1i480co.pdf Recruitment Guidance for additional information.

Chart review by the PI will be performed to determine potential subjects coming into the outpatient surgery clinic. Surgeons, nurse practictioners, physician assistants, and nurses who are directly taking care of the patient will ask eligible patients during that surgical consultation visit whether they are interested in enrolling/hearing more about the study. If they are, they will be referred to the research staff who will either

enroll them in person or over the phone.

h) Inclusion and Exclusion Criteria.

Identify inclusion criteria.

Adult patients aged ≥ 18 years undergoing major elective surgeries, under general anaesthesia (e.g.,colon or colorectal resection, partial or total gastrectomy, pancreaticoduodenectomy, hepatectomy, cystectomy or major gynecologic, thoracic, plastic and neurosurgeries)

Identify exclusion criteria.

Children (<18 years), patients undergoing emergency or urgent surgery
Patients undergoing surgery in <28 days, non-english speakers will not be included. Premorbid conditions or orthopedic impairments that contraindicated physical exercise, cognitive disabilities, ASA score 4 or higher or patient under palliative care, expected length of stay at hospital < 48 hours and no access to internet will be also criteria of non inclusion.

i) Describe your screening procedures, including how qualifying laboratory values will be obtained. If you are collecting personal health information prior to enrollment (e.g., telephone screening), please request a waiver of authorization for recruitment (in section 15).

Screening will be performed by the PD who will screen the electronic medical records of the patients to be seen in the clinics of the colorectal surgeons to identify potential participants. A waiver of authorization for recruitment will be requested.

j) Describe how you will be cognizant of other protocols in which participants might be enrolled. Please explain if participants will be enrolled in more than one study.

We will ask the patients if they are in any other protocols (except the protocol IRB #46978 in which they could be included). They will be allowed to be enrolled in other studies as long as they do not interfere with this study (for example, they may be enrolled in Tissue Bank or GenePool, but not in any other rehabilitation, diet, or exercise studies).

k) Payment/reimbursement. Explain the amount and schedule of payment or reimbursement, if any, that will be paid for participation in the study. Substantiate that proposed payments are reasonable and commensurate with the expected contributions of participants and that they do not constitute undue pressure on participants to volunteer for the research study. Include provisions for prorating payment. See payment considerations

There will be no monetary compensation for the study.

l) Costs. Please explain any costs that will be charged to the participant.

There will be no costs that will be charged to the participants.

m) Estimate the probable duration of the entire study. Also estimate the total time per participant for: (i) screening of participant; (ii) active participation in study; (iii) analysis of participant data.

Probable duration of the entire study: 6 months

Per participant:
(i) screening: 15 minutes
(ii) active participation in study: 90 days
(iii) analysis of data: 1 hour

9. Risks

a) For the following categories include a scientific estimate of the frequency, severity, and reversibility of potential risks. Wherever possible, include statistical incidence of complications and the mortality rate of proposed procedures. Where there has been insufficient time to accumulate significant data on risk, a statement to this

effect should be included. (In describing these risks in the consent form to the participant it is helpful to use comparisons which are meaningful to persons unfamiliar with medical terminology.)

The risks of the Investigational devices.

None

The risks of the Investigational drugs. Information about risks can often be found in the Investigator's brochure.

none

The risks of the Commercially available drugs, reagents or chemicals. Information about risks can often be found in the package insert.

none

The risks of the Procedures to be performed. Include all investigational, non-investigational and non-invasive procedures (e.g., surgery, blood draws, treadmill tests).

Investigational and procedures: Physical therapy and surveys. The risk associated with these procedures is low to null as the study protocol involves exercises to be done only at the comfort level of the patient. Blood draws will be collected by licensed personnel using sterile supplies and aseptic technique (as validated by the IRB-#46978)

Non-investigational: elective surgery and associated post-operative care, and pre-operative testing. The risk of these procedures varies according to the procedure and the patient, but are not expected to increase as a result of the study activities.

The risks of the Radioisotopes/radiation-producing machines (e.g., X-rays, CT scans, fluoroscopy) and associated risks.

none

The risks of the Physical well-being.

There are minimal risks to the physical well-being of the participants, as the study protocol involves exercises to be done only at the comfort level of the patient, and well-accepted dietary, cognitive or well-being recommendations.

The risks of the Psychological well-being.

There are minimal risks to the psychological well-being of the participants.

The risks of the Economic well-being.

There are no risks to the economic well-being of the participants as the study protocol will not incur additional costs to the patients.

The risks of the Social well-being.

There are no risks to the social well-being of the participants as the study protocol will not involve their social situation.

b) If you are conducting international research, describe the qualifications/preparations that enable you to both estimate and minimize risks to participants. Provide an explanation as to why the research must be completed at this location and complete the International Research Form. If not applicable, enter N/A.

none

c) Describe the planned procedures for protecting against and minimizing all potential risks. Include the means for monitoring to detect hazards to the participant (and/or to a potential fetus if applicable). Include steps to minimize risks to the confidentiality of identifiable information.

Administration of frequent surveys to detect increase in stress and pain levels will monitor for hazards. Also, patients will be closely monitored in the post-operative inpatient period for any physical problems.

The confidentiality of identifiable information will be protected as only the members of the study staff will

have access to the information, and the data will be stored on a secure computer (the PI's computer) which is password protected, encrypted by Stanford IT, and located in a locked office in a building under 24-hour surveillance.

d) Explain the point at which the experiment will terminate. If appropriate, include the standards for the termination of the participation of the individual participant Also discuss plans for ensuring necessary medical or professional intervention in the event of adverse effects to the participants.

The study poses only a small risk to participants. There is no plan to discontinue the study before all participants have been enrolled and samples have been collected. Drs. Kin and Gaudilliere will be available in the unlikely event that a participant needs medical attention as a result of study participation.

Individual participants may be terminated from the study if they choose to no longer be in the study.

In the event of adverse effects to the participants, the study staff will ensure that they receive the necessary medical and professional intervention.

10. Benefits

a) Describe the potential benefit(s) to be gained by the participants or by the acquisition of important knowledge which may benefit future participants, etc.

The potential benefits to be gained by the participants include
increased fitness, improved nutritional and cognitive status, stress and
pain reduction and improved outcomes / patient experience after surgery

11. Privacy and Confidentiality

Privacy Protections

a) Describe the setting and method (e.g. crowded waiting room, patient exam room, telephone or email communication) in which interactions will occur and how the privacy interests of participants will be maintained. Note, high risk data such as PHI must be sent via "Secure:" email per https://uit.stanford.edu/security/hipaa/email-policy Stanford policy.

Phone calls will be conducted in a standard manner to confirm the
identity of the participant, and will be initiated only by a member of
the study staff.
In-person interactions will occur in locations that offer privacy for
the participants.
Care will always be taken to preserve the patients privacy during
discussion.

Confidentiality Protections

b) Specify PHI (Protected Health Information). PHI is health information linked to HIPAA identifiers (see above). List BOTH health information AND HIPAA identifiers. If you are using STARR, use the Data Privacy Attestation to ensure that your request will match your IRB-approved protocol. Be consistent with information entered in section 15a.

HIPAA identifiers: Names, telephone numbers, emails, dates, medical
record numbers, address
Health information: Age, gender, diagnosis, operation, surgeon, date of
operation, date of discharge, date of bowel function return, narcotic
use post-op, results of study surveys, self-reported physical activity
and dietary adherence, surgical complications.

c) You are required to comply with University Policy that states that ALL electronic devices: computers (laptops and desktops; OFFICE or HOME); smart phones; tablets; external hard disks, USB drives, etc. that may hold identifiable participant data will be password protected, backed up, and encrypted. See http://med.stanford.edu/datasecurity/ for more information on the Data Security Policy and links to encrypt your devices.

Stanford University IT approved platforms (https://uit.stanford.edu/guide/riskclassifications https://uit.stanford.edu/guide/riskclassifications) should be used for data management. Consult with your Department IT representative for more information.For data security policies and links to encrypt your devices see http://med.stanford.edu/irt/security and target=_blankhttp://www.stanford.edu/group/security/securecomputing/mobile_devices.html. Additionally, any PHI data on paper must be secured in a locked environment.

By checking this box, You affirm the aforementioned. Y

RedCap will be used. If patients prefer to take surveys on paper forms, then the research staff will enter the data into RedCap immediately and then destroy the hard copies.

d) Describe how data or specimens will be labeled (e.g. name, medical record number, study number, linked coding system) or de-identified. If you are de-identifying data or specimens, who will be responsible for the de-identification? If x-rays or other digital images are used, explain how and by whom the images will be de-identified.

Data will be labeled with name and study number.

e) Indicate who will have access to the data or specimens (e.g., research team, sponsors, consultants) and describe levels of access control (e.g., restricted access for certain persons or groups, access to linked data or specimens).

Only the PI and Research staff will have access to the data.

f) If data or specimens will be coded, describe the method in which they will be coded so that study participants' identities cannot be readily ascertained from the code.

NA

g) If data or specimens will be coded, indicate who will maintain the key to the code and describe how it will be protected against unauthorized access.

NA

h) If you will be sharing data with others, describe how data will be transferred (e.g., courier, mail) or transmitted (e.g., file transfer software, file sharing, email). If transmitted via electronic networks, describe how you will secure the data while in transit.See http://www.stanford.edu/group/security/securecomputing/. http://www.stanford.edu/group/security/securecomputing/. Additionally, if you will be using or sharing PHI see https://uit.stanford.edu/security/hipaa https://uit.stanford.edu/security/hipaa.

Data will not be transferred electronically. The data will remain on RedCap.

i) How will you educate research staff to ensure they take appropriate measures to protect the privacy of participants and the confidentiality of data or specimens collected (e.g. conscious of oral and written communications, conducting insurance billing, and maintaining paper and electronic data)?

Research staff will be educated on confidentiality of data and how to maintain utmost privacy for participants at all times.

12. Potential Conflict of Interest

Title : Immune system modulation by enhanced vs standard prehabilitation program in patients undergoing elective major surgery - a prospective monocentric randomized single-blinded control . . .

Approval Period: 10/23/2023 - 12/31/2999

Investigators are required to disclose any outside interests that reasonably appear to be related/li to this protocol.

Outside Interest Tasks

Investigators	Role	Potential COI?	Date Outside Interest Answered	Date OPACS Disclosure Submitted	COI Review Determination
Cindy Kin	PD	N	07/04/2020		N/A
Brice Louis Jules Gaudilliere	COPD	N	07/04/2020		N/A
Dyani Kalea Gaudilliere	OP	N	07/05/2020		N/A

13. Consent Background

13. 1 Consent Prehab_Surge_Consent_V6

Sponsor's Consent Version Number: (if any) :

a) Describe the informed consent process. Include the following.

- **i) Who is obtaining consent? (The person obtaining consent must be knowledgeable about the study.)**
- **ii) When and where will consent be obtained?**
- **iii) How much time will be devoted to consent discussion?**
- **iv) Will these periods provide sufficient opportunity for the participant to consider whether or not to participate and sign the written consent?**
- **v) What steps are you taking to minimize the possibility of coercion and undue influence?**
- **vi) If consent relates to children and if you have a reason for only one parent signing, provide that rationale for IRB consideration.**

i) The research coordinator, other member of the research team, or clinician trained in the study procedures and consent process will obtain the consent. ii) The consent will be obtained in the surgical clinic before their scheduled surgery. In response to a desire to limit in person contact post COVID-19, when possible we will obtain consent remotely via phone/zoom. In these cases a consent signature may be obtained using REDCap. iii) As much time as is needed for thorough understanding of the consent and implications of participation will be taken. iv) Yes v) All persons obtaining consent will be informed of the need to ensure participants are not coerced when obtaining consent. All participants will be informed that participation is entirely voluntary and that their decision will not effect their clinical care. vi) NA

b) What is the Procedure to assess understanding of the information contained in the consent? How will the information be provided to participants if they do not understand English or if they have a hearing impairment? See HRPP Chapter12.2 for guidance.

Participants will be instructed to ask questions if they do not understand. If there is suspicion that a participant does not understand, questions will be asked of them by the research staff member obtaining consent to determine the degree of comprehension. Participants who do not understand English won't be included.

Title : Immune system modulation by enhanced vs standard prehabilitation program in patients undergoing elective major surgery - a prospective monocentric randomized single-blinded control . . .

Approval Period: 10/23/2023 - 12/31/2999

c) What steps are you taking to determine that potential participants have the capacity to participate in the decision-making process? If your study may enroll adults who are unable to consent, describe (i) how you will assess the capacity to consent, (ii) what provisions will be taken if the participant regains the capacity to consent,(iii) who will be used as a legally authorized representative, and (iv) what provisions will be made for the assent of the participant.

i)The participants will be evaluated by a study team member to confirm competence. ii), iii) and iv) The study will not enroll people who are unable to consent. If the patient is competent to participate in the decision-making process to undergo elective surgery, then they are eligible for this study.

14. Assent Background (less than 18 years of age)

15. HIPAA Background

15. 1 Waiver of Authorization for Recruitment prehab_surge_waiver

a) Describe the protected health information (PHI) needed to conduct screening or recruitment. PHI is health information linked to HIPAA identifiers. List BOTH health information AND HIPAA identifiers. If you are using STARR, use the Data Privacy Attestation to ensure that your request will match your IRB-approved protocol.

Age, type of operation planned, date of operation.

b) Please Answer:

Y **Do you certify that the use or disclosure of protected health information involves no more than a minimal risk to the privacy of individuals?**

Y **Do you certify that the research could not practically be conducted with out the waiver?**

Y **Do you certify that you have adequate written assurances that the protected health information will not be reused or disclosed to any other person or entity, except as required by law, for authorized oversight of the research project, or for other research for which the use or disclosure of protected health information would be permitted?**

Y **Do you certify that the research could not practically be conducted with out access to and use of the protected health information?**

c) Please describe an adequate plan to protect any identifiers from improper use and disclosure.

Data will be stored on REDCap. Only study personnel will have access to the data.

d) Please describe an adequate plan to destroy the identifiers at the earliest opportunity consistent with conduct of the research, unless there is a health or research justification for retaining the identifiers or such retention is otherwise required by law.

Identifiers will be destroyed when data collection and data analysis are complete.

16. Attachments

Attachment Name	Attached Date	Attached By	Submitted Date
ControlGroup_MedDietResources	07/23/2020	fverdonk	

ControlGroup_PhysicalExerciseList	07/23/2020	fverdonk	
qMCI	07/23/2020	fverdonk	
SF36_scale	07/23/2020	fverdonk	
AmsterdamAnxiety	07/23/2020	fverdonk	
PainCastraphScale4	07/23/2020	fverdonk	
MedDiet_Scale	07/23/2020	fverdonk	
Pain_DN4_scale	07/24/2020	fverdonk	
ControlGroup_ProgramBook	07/27/2020	fverdonk	
Compliance_scale	07/27/2020	fverdonk	
LUMOSITY_EndUserLicenseAgreement	08/26/2020	fverdonk	
LUMOSITY_PrivacyPolicy	08/26/2020	fverdonk	
TIMEDWALK_PrivacyPolicy	08/26/2020	fverdonk	
DRA_answer	08/26/2020	fverdonk	
SSV1	09/15/2020	agoel247	
e-consent_V1	11/18/2020	fverdonk	

Obligations

The Protocol Director agrees to:

- Adhere to principles of sound scientific research designed to yield valid results
- Conduct the study according to the protocol approved by the IRB
- Be appropriately qualified to conduct the research and be trained in Human Research protection, ethical principles, regulations, policies and procedures
- Ensure all Stanford research personnel are adequately trained and supervised
- Ensure that the rights and welfare of participants are protected including privacy and confidentiality of data
- Ensure that, when de-identified materials are obtained for research purposes, no attempt will be made to re-identify them.
- Disclose to the appropriate entities any potential conflict of interest
- Report promptly any new information, modification, or unanticipated problems that raise risks to participants or others
- Apply relevant professional standards.

Any change in the research protocol must be submitted to the IRB for review prior to the implementation of such change. Any complications in participants or evidence of increase in the original estimate of risk should be reported at once to the IRB before continuing with the project. Inasmuch as the Institutional Review

Board (IRB) includes faculty, staff, legal counsel, public members, and students, protocols should be written in language that can be understood by all Panel members. The investigators must inform the participants of any significant new knowledge obtained during the course of the research.

IRB approval of any project is for a maximum period of one year. For continuing projects and activities, it is the responsibility of the investigator(s) to resubmit the project to the IRB for review and re-approval prior to the end of the approval period. A Notice to Renew Protocol is sent to the Protocol Director 7 weeks prior to the expiration date of the protocol.

https://stanfordmedicine.box.com/shared/static/qbsi8u8h47qsotxhdpuzz50xlrqa0sgo.pdf Report promptly any new information, complaints, possibly serious and/or continuing noncompliance, or unanticipated problems involving risks to participants or others.

All data including signed consent form documents must be retained for a minimum of three years past the completion of the research. Additional requirements may be imposed by your funding agency, your department, or other entities. (Policy on Retention of and Access to Research Data, Research Policy Handbook, http://doresearch.stanford.edu/policies/research-policy-handbook/conduct-research/retention-and-access-research-data)

APPROVAL LETTER/NOTICE NOTE: List all items (verbatim) that you want to be included in your approval letter (e.g., Amendment date, Investigator's Brochure version, consent form(s) version(s), advertisement name, etc.) in the box below.

Y By checking this box, I verify that I, as the Protocol Director (PD) responsible for this research protocol, have read and agree to abide by the above obligations, or that I have been delegated authority by the PD to certify that the PD has read and agrees to abide by the above obligations.
