# Supplementary material for: Immune Modulation by Personalized vs Standard Prehabilitation Before Major Surgery: A Randomized Clinical Trial
Source: JAMA Surg. 2025 Nov 12;161(1):20–30. doi: 10.1001/jamasurg.2025.4917 (PMC12613092; doi:10.1001/jamasurg.2025.4917)
Supplement: Supplement 2. — eMethods eFigure 1. Immune cell gating strategy eFigure 2. Study design and workflow eFigure 3. Immune distribution and signaling response before and after prehab eTable 1. Mass Cytometry antibody panel eTable 2. adherence score per specific prehab domain eTable 3. Postoperative outcomes eTable 4. Evolution of major cell type frequencies after prehab [file jamasurg-e254917-s002.pdf]

Supplemental Online Content

Cambriel A, Tsai A, Choisy B, et al. Immune modulation by personalized vs standard prehabilitation before major surgery: a randomized clinical trial. *JAMA Surgery*. Published online November 12, 2025. doi:10.1001/jamasurg.2025.4917

eMethods

eFigure 1. Immune cell gating strategy

eFigure 2. Study design and workflow

eFigure 3. Immune distribution and signaling response before and after prehab

eTable 1. Mass Cytometry antibody panel

eTable 2. adherence score per specific prehab domain

eTable 3. Postoperative outcomes

eTable 4. Evolution of major cell type frequencies after prehab

This supplemental material has been provided by the authors to give readers additional information about their work.

© 2025 Cambriel A et al. *JAMA Surgery*.

eMethods:

Prehab programs

The **Notion® platform** served as a centralized digital interface customized for each participant in the personalized arm. A personalized page was shared with the patient and served as a backbone for the prehab program. It was developed to support engagement across the four prehabilitation pillars — nutrition, physical activity, mindfulness, and cognition — and offered the following features:

- **Structured daily content**: The platform included a 21-day **mindfulness meditation course**, with short (10-15 minute) guided audio sessions organized into three progressive thematic weeks: (1) body awareness and breathing, (2) visualization, and (3) positive anticipation. Sessions were linked directly within the platform for easy access.
- **Physical activity support**: Weekly exercise sessions were delivered via embedded infographics created by a doctor in physical therapy. These included bodyweights strengthening, mobility drills, aerobic and anaerobic activity, adapted to each patient's functional level (based on the SPPB and 30s Chair Stand test). Patients were encouraged to repeat sessions 3–5 times per week.
- **Nutritional guidance**: though the Notion platform, a personalized nutrition plan was presented visually, including a list of recommended foods (based on the Mediterranean diet), daily hydration goals, and sample recipes. Patients could self-track dietary intake using a daily checklist and visualize adherence with a weekly compliance dashboard embedded in the page.
- **Self-monitoring and engagement**: Interactive checklists allowed patients to log daily behaviors related to all four pillars. These indicative visuals used by both the patient and the coach to monitor progress and tailor support during weekly video calls coaching sessions. This system enabled individualized feedback, motivational adjustments, and dynamic prioritization of goals.

The **Lumosity® app** was used to support cognitive training. Lumosity is a commercially available digital platform that delivers short, game-based tasks targeting memory, attention, executive function, and processing speed. Participants were instructed to complete a daily 10–15-minute session through the app. Performance metrics were visible to the user, and patients were prompted via Notion to maintain daily use. Compliance was reviewed weekly with the coach through verbal check-in and self-reported entries. The use of the Lumosity® app for cognitive prehabilitation was supported by prior evidence demonstrating that preoperative cognitive training significantly reduced postoperative delirium incidence in older adults undergoing major non-cardiac surgery.[1]

Adherence evaluation

Patients were called weekly by a trained Clinical Research Coordinator (ES) who was blind to group assignments. They were asked the following questions:

- How would you describe the frequency with which you followed the prehabilitation plan this week?
- How many days did you train with the physical exercises this week?
- How many days did you strictly follow the nutritional recommendations this week?
- How many days did you strictly follow the mindfulness recommendation this week?

Responses were scored with a Likert scale from 1 to 4: 1 =Never; 2= Occasionally or once to twice a week; 3= Often or 3 to 6 times a week; 4= Always or 7 times a week.

- Would you describe your attitude toward the prehabilitation process as positive this week?
- Would you say that you've given 100% effort in prehabilitation process this week?
- Would you say that you were motivated to complete the prehabilitation process this week?

Responses were scored with a Likert scale ranging from 1 to 4; 1= strongly disagree, 2= disagree, 3= neutral, 4= Agree.

© 2025 Cambriel A et al. *JAMA Surgery*.

Single cell analysis

Blood collection

Whole blood samples were collected at enrollment, i.e. before prehab, and on the day of surgery before anesthesia. They were collected in sodium heparinized tubes. Then, samples were either left unstimulated to measure endogenous intracellular activities or exposed to either the toll like receptor (TLR) 4 agonist lipopolysaccharide (LPS), or Interleukine (IL) 2,4 and 6, or Tumor necrosis factor alpha (TNFα) to activate canonical surgical trauma related signaling responses in vitro. This in vitro *immune stress test* aims to replicate surgical trauma in vitro and provide an in depth analysis of patients' immunome[2,3] (**Figure 1B**). All samples were then fixed with Proteomic Stabilizer in Smart Tubes (Smart α Inc., San Carlos, CA) and immediately stored at -80°C.

Barcoding, antibody staining and mass cytometry processing

Following erythrocyte lysis, samples were barcoded and stained with both surface and intracellular antibodies according to standard protocols[4,5]. The antibody panel comprised 41-marker including 26 antibodies targeting cell-surface markers and 15 intracellular antibodies specific to phosphorylated (p) signaling epitopes (**Table S1**). The selection of immune cell types and intracellular signaling pathways was informed by previous studies on the peripheral immune response to surgery [2,4,6] and preclinical evidence related to immune processes involved in post-operative complications, particularly surgical site infection (SSI) and postoperative neurocognitive decline (POND) [7]. To reduce variability in measurement, samples from each individual patient for a specific stimulation condition were processed simultaneously. The resulting FCS files were normalized and de-barcoded using MatLab-based software, then uploaded to the Cell Engine platform (https://cellengine.com, Primity Bio, Fremont, CA) for manual gating.

Cell frequency, endogenous intracellular signaling, and intracellular signaling responses

For each patient, we analyzed 1096 single-cell proteomic features per timepoint. Features included the frequency of 41 key innate and adaptive immune cells defined through manual gating based on pre-established gating strategies [4] (**Fig. S1**), as well as their intracellular signaling activities (e.g., the phosphorylation state of 11 proteins including pSTAT1, pSTAT3, pSTAT5, pSTAT6, pNF-κB, pMAPKAPK2, pP38, prpS6, pERK1/2, pCREB, and total IκBα). Immune cell frequency features were calculated for each immune cell subset from the unstimulated samples. For each cell type, intracellular activities were reported as the median signal intensity (arcsinh transformed value). Changes in signaling in response to receptor-specific ligands were expressed as the arcsinh transformed ratio relative to the endogenous signaling, i.e., the difference in arcsinh transformed signal intensity between the stimulated and unstimulated condition. The proportion of mononuclear cells was reported as a percentage of the gated live mononuclear cells. The proportion of neutrophils was reported as a percentage of the gated live leukocytes.

Preprocessing and Visualization of the single-cell proteomic data set

Before conducting statistical analysis, three preprocessing steps were applied to the initial dataset. First, a knowledge-based penalization matrix was applied to the intracellular signaling response features in the mass cytometry data based on mechanistic immunological knowledge, as previously described[2]. Second, features that exhibited no variance were excluded from the dataset. Lastly, features were Z-scored before fitting any model. The single-cell mass cytometry dataset was then visualized using a uniform manifold approximation and projection (UMAP) layout.

Stabl algorithm

Stabl is a supervised machine learning framework that allows for sparse, reliable, and predictive feature selection[8]. Stabl is particularly well-suited for this study, given the relatively small number of observations (n) compared to the number of features (p). It yields a sparse solution that builds the final model from a limited number of selected features. Here, a Lasso model with 500 bootstraps with replacement iteration was used. Missing data was treated with median imputation. The noise injection strategy was knockoff and is designed to mimic the statistical structure of the input

© 2025 Cambriel A et al. *JAMA Surgery*.

data, including correlations between samples. This implicitly models the dependencies between paired measurements (e.g., pre/post samples from the same patient) during stability selection, thereby mitigating overfitting individual patient trajectories. The artificial proportion of injected data was 1. The optimal threshold used for each layer was data-driven. During cross-validation, we employed a leave-one-patient-out (LOPO) cross-validation strategy to prevent data leakage and ensure independence between training and test sets. This guarantees that both pre- and post-intervention samples from the same patient never co-occur in training and validation folds. The model predictions over the test data within the cross-validation folds were then assessed via the AUROC (Area Under the Receiver Operating Characteristic), with statistical significance being calculated via Mann-Whitney U-test p-values on these predictions, with a $p\text{-value} < 0.05$ considered statistically significant.

The package for Stabl is available online.

© 2025 Cambriel A et al. *JAMA Surgery*.

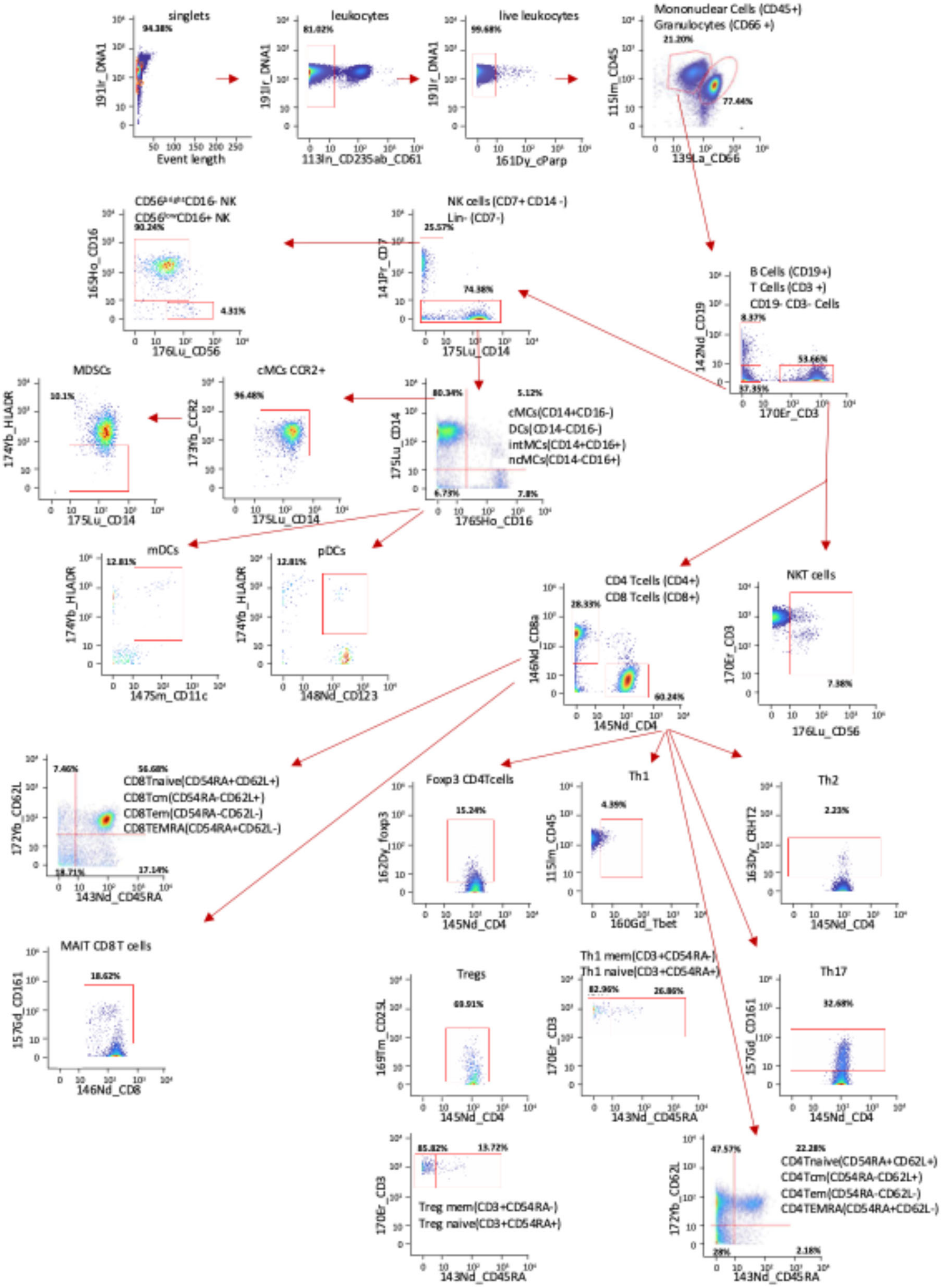

eFigure 1: Immune cell gating strategy

© 2025 Cambriel A et al. *JAMA Surgery*.

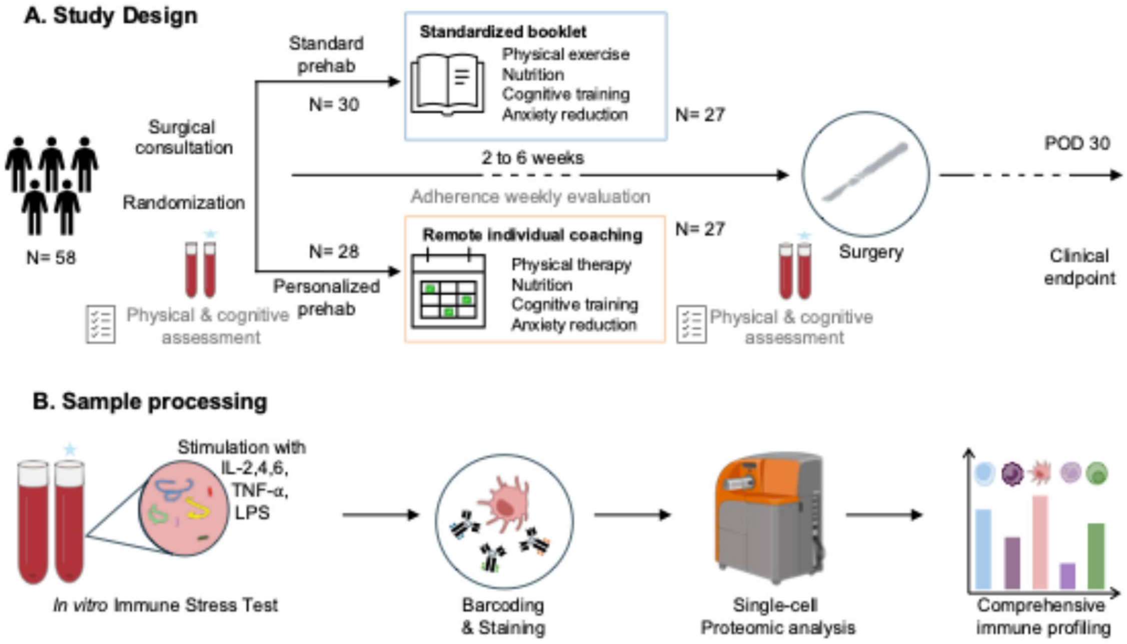

eFigure 2: Study design and workflow. A. Study design: 58 patients undergoing major surgery were randomized to receive either standard prehabilitation (prehab) (n=30) or personalized prehab (n=28) at the preoperative surgical consultation. Clinical evaluation and blood sample collection was performed at two time points: at time of randomization (pre-prehab) and preoperatively (post-prehab). Prehab lasted 2-6 weeks, and adherence was evaluated weekly. 27 patients from each arm completed prehab and were included in the final analysis. Clinical endpoints included complications assessed within 30 postoperative days (POD) using the Clavien-Dindo classification and hospital length of stay. **B.** Sample processing: whole blood samples were either left unstimulated to measure endogenous intracellular activities or exogenously stimulated with toll-like receptor-4 agonist lipopolysaccharide (LPS), Interleukins (IL)-2,4,6, or tumor necrosis factor-alpha (TNF-α) to activate canonical pathways in a surgical trauma model and provide an in-depth analysis of patients' immunomes.[33,61] Following erythrocyte lysis, samples were barcoded and stained with both surface and intracellular antibodies according to standard protocols and assessed simultaneously to minimize experimental variability.[26,27] Samples were measured using a 47-multiplex mass cytometry assay yielding a highly comprehensive immune profile for each patient.

This figure was created using elements from BioRender.com.

.

© 2025 Cambriel A et al. *JAMA Surgery*.

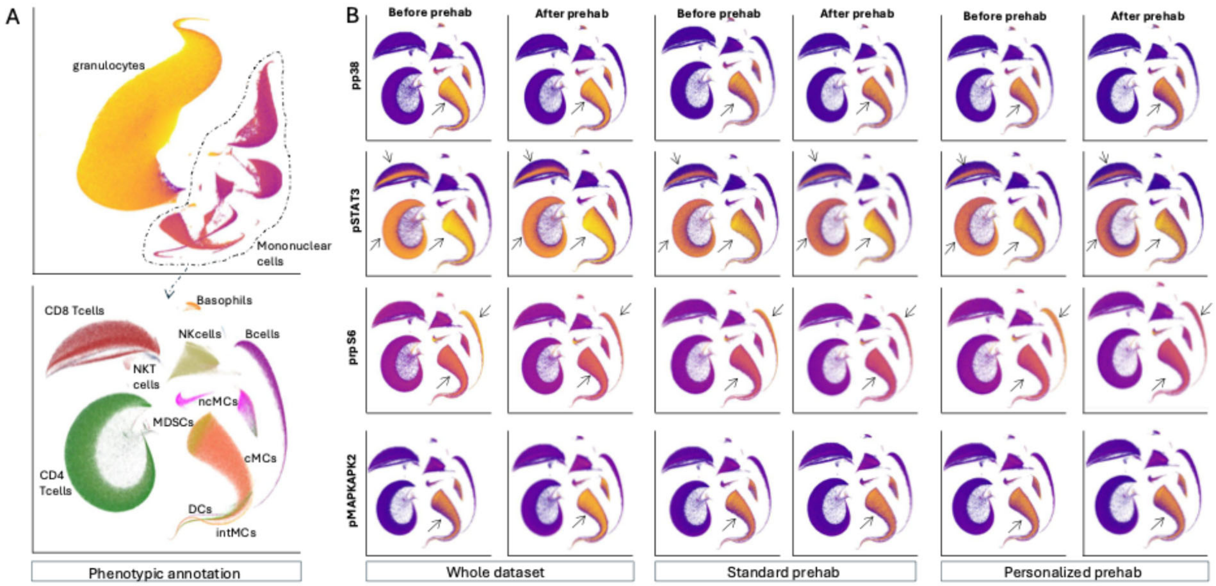

eFigure 3: Immune distribution and signaling response before and after prehab A. UMAP representation of the single-cell mass cytometry dataset. Top, all live leukocytes, including neutrophils and mononuclear cells; Bottom: UMAP representation of mononuclear cells only. UMAPS are clustered by cell types and annotated. **B.** UMAPs representing all mononuclear cells colored according intra-cellular signaling before and after prehab; left for the whole dataset, middle for the standard prehab group, right for the personalized prehab group *Abbreviations: cMCs: classical monocytes; CREB: cAMP-response element binding protein; intMCs: intermediate monocytes; mDCs: myeloid dendritic cells; MAPKAPK2: Mitogen-activated protein kinase-activated protein kinase ; MDSCs: Myeloid Derived Suppressor Cells; NF-κB: Nuclear factor – κB; ncMCs: non classical monocytes; NK cells: natural killer cells; pDCs: plasmacytoid dendritic cells; p: phosphorylation; STAT: Janus Kinase/signal transducers and activators of transcriptions; TNFα: Tumor Necrosis Factor alpha*

.

© 2025 Cambriel A et al. *JAMA Surgery*.

Antibody	Manufacturer	Metal	Isotope	Clone	Concentration	Comment
**Barcode1**	Trace Sciences	Pd	102		15µM	Barcode
**Barcode2**	Trace Sciences	Pd	104		15µM	Barcode
**Barcode3**	Trace Sciences	Pd	105		15µM	Barcode
**Barcode4**	Trace Sciences	Pd	106		15µM	Barcode
**Barcode5**	Trace Sciences	Pd	108		15µM	Barcode
**Barcode6**	Trace Sciences	Pd	110		15µM	Barcode
**CD235ab**	Biolegend	In	113	HIR2	1µg/mL	Phenotype
**CD61**	BD	In	113	VI-PL2	0.5µg/mL	Phenotype
**CD45**	Biolegend	In	115	HI30	1µg/mL	Phenotype
**CD66**	BD	La	139	CD66a-B1.1	0.5µg/mL	Phenotype
**CD7**	BD	Pr	141	M-T701	0.5µg/mL	Phenotype
**CD19**	Biolegend	Nd	142	HIB19	0.5µg/mL	Phenotype
**CD45RA**	Biolegend	Nd	143	HI100	0.5µg/mL	Phenotype
**CD11b**	Biolegend	Nd	144	ICRF44	2µg/mL	Phenotype
**CD4**	Biolegend	Nd	145	RPA-T4	2µg/mL	Phenotype
**CD8a**	Biolegend	Nd	146	RPA-T8	1µg/mL	Phenotype
**CD11c**	Biolegend	Sm	147	Bu15	1µg/mL	Phenotype
**CD123**	Biolegend	Nd	148	6H6	1µg/mL	Phenotype
**pCREB**	Cell Signaling Technology	Sm	149	87G3	2µg/mL	Function
**pSTAT5**	Cell Signaling Technology	Nd	150	C11C5	4µg/mL	Function
**pP38**	BD	Eu	151	36/p38	2µg/mL	Function
**TCRgd**	BD	Sm	152	B1	4µg/mL	Phenotype
**pSTAT1**	BD	Eu	153	14/P-STAT1	1µg/mL	Function
**pSTAT3**	Cell Signaling Technology	Sm	154	M9C6	2µg/mL	Function
**pS6**	Cell Signaling Technology	Gd	155	D57.2.2E	2µg/mL	Function
**FceRI**	Biolegend	Gd	156	AER-37 (CRA-1)	0.5 µg/mL	Phenotype
**CD33**	Biolegend	Gd	158	WM53	2µg/mL	Phenotype
**pMAPKAPK2**	Cell Signaling Technology	Tb	159	27B7	1µg/mL	Function
**Tbet**	Thermo Fisher	Gd	160	4B10	8µg/mL	Phenotype
**cPARP**	BD	Dy	161	F21-852	1µg/mL	Phenotype
**FoxP3**	Thermo Fisher	Dy	162	PCH101	8µg/mL	Phenotype
**IκB**	Cell Signaling Technology	Dy	164	L35A5	8µg/mL	Function
**CD16**	Biolegend	Ho	165	3G8	1µg/mL	Phenotype
**pNF-κB**	BD	Er	166	K10-895.12.50	2µg/mL	Function
**pERK1-2**	Cell Signaling Technology	Er	167	D13.14.4E	4µg/mL	Function
**pSTAT6**	Biolegend	Er	168	A15137E	1µg/mL	Function
**CD25**	Biolegend	Tm	169	M-A251	2µg/mL	Phenotype
**CD3**	Biolegend	Er	170	UCHT1	1µg/mL	Phenotype
**CXCR4**	BD	Yb	171	M-T271	2µg/mL	Phenotype
**CD62L**	Biolegend	Yb	172	W6D3	0.5µg/mL	Phenotype
**CCR2**	Biolegend	Yb	173	K036C2	2µg/mL	Phenotype
**HLA-DR**	Fluidigm	Yb	174	L243	2µg/mL	Phenotype
**CD14**	Fluidigm	Lu	175	M5E2	2µg/mL	Phenotype
**CD56**	BD	Lu	176	NCAM16.2	1µg/mL	Phenotype
**DNA1**	Fluidigm	Ir	191		50µM	DNA
**DNA2**	Fluidigm	Ir	193		50µM	DNA

eTable 1: Mass Cytometry antibody panel

© 2025 Cambriel A et al. *JAMA Surgery*.

Adherence domain	Standard prehab	Personalized prehab	P value
Physical exercises	3.1 (0.8)	3.1 (0.5)	0.8628476
Nutrition	3.3 (0.8)	3.6 (0.4)	0.29084441
Mindfulness	2.8(1.2)	3.3 (0.7)	0.49487108
Positive attitude toward prehab	3.9(0.3)	3.9 (0.3)	0.5425202
100% effort towards prehab	3.5(0.7)	3.6 (0.5)	0.9683208
Motivation towards prehab process	3.7(0.5)	3.9 (0.1)	0.15756785

Results are expressed as mean average adherence over the prehab duration and standard deviation.

eTable 2: adherence score per specific prehab domain

© 2025 Cambriel A et al. *JAMA Surgery*.

	Standard prehab N=27	Personalized prehab N=27	p
**Patients with Clavien-Dindo complication grade >1**	11 (40.7)	4 (15.8)	**0.037**[1]
**Complication grade at POD30 according to Clavien-Dindo classification**			
Grade 0	0 (0)	2 (7.1)	
Grade 1	18 (64.3)	21 (75.0)	
Grade 2	8(28.6)	3(10.7)	
Grade 3a	2(7.1)	0 (0)	
Grade 3b	1(3.5)	1 (3.5)	
**Hospital length of stay, days**	2.6[1.5,6.03]	2.12[0.66,4.02]	0.48[2]

[1]Mann-Whitney Utest

[2]Wilcoxon rank sum test

eTable 3: Postoperative outcomes

© 2025 Cambriel A et al. *JAMA Surgery*.

Cell type frequency	Standard prehab		Personalized prehab	
	Before prehab	After prehab	Before Prehab	After prehab
Granulocytes	69.3 [61.9,76.6]	67.7[60.8,75.9]	69.2 [61.6,74.7]	71.4[62.4,76.0]
cMCs	19.4 [14.3,27.6]	26.5[17.3,34.2]	20.6 [15.4,27.48]	23.9[18.5,27.6]
T cells	53.12 [45.5,63.6]	52.4[42.4,59.2]	54.5[50.0,61.3]	52.8[45.3,57.8]
B cells	5.4 [3.7,7.2]	4.6[2.7,6.2]	6.3 [2.6,7.5]	5.1[2.7,6.9]

Frequencies of granulocytes are expressed as percentages of all leukocytes, frequencies of classical monocytes (cMCs), T and B cells are expressed as percentages of mononuclear cells.

eTable 4: Evolution of major cell type frequencies after prehab.

© 2025 Cambriel A et al. *JAMA Surgery*.
