# Supplementary material for: Immune Modulation by Personalized vs Standard Prehabilitation Before Major Surgery: A Randomized Clinical Trial
Source: JAMA Surg. 2025 Nov 12;161(1):20–30. doi: 10.1001/jamasurg.2025.4917 (PMC12613092; doi:10.1001/jamasurg.2025.4917)
Supplement: Supplement 3. — Data sharing statement [file jamasurg-e254917-s003.pdf]

Data Sharing Statement

Cambriel. Immune Modulation by Personalized vs Standard Prehabilitation Before Major Surgery. *JAMA Surg*. Published November 12, 2025. doi:10.1001/jamasurg.2025.4917

Data
Additional Information: ClinicalTrial.gov, https://clinicaltrials.gov/study/NCT04498208?term=gaudilliere&rank=1, NCT04498208
Data available: Yes
Data types: Deidentified participant data, Data dictionary
How to access data: gbrice@stanford.edu
When available: With publication

Supporting Documents
Document types: Statistical/analytic code
How to access documents: gbrice@stanford.edu
When available: With publication

Additional Information
Who can access the data: anyone requesting the data
Types of analyses: for any purpose
Mechanisms of data availability: after approval of a proposal
